# Artificial intelligence in rehabilitation: a review of clinical effectiveness, real-world performance, safety, and equity across modalities and settings

**DOI:** 10.3389/fdgth.2026.1737957

**Published:** 2026-03-18

**Authors:** Nafisa Abdalla, Rabie Adel El Arab, Amany Abdrbo, Mohammed Almari, Mohammed Yahya Ayoub, Bilal Alsaaideh, Mohammad Suhail Dagamseh, Wesam Taher Almagharbeh, Fuad Abuadas, Mohammad S. Abu Mahfouz, Mastoura Khames Gaballah

**Affiliations:** 1 Almoosa College of Health Sciences, Alhsa, Saudi Arabia; 2 Dr. Sulaiman Al Habib Medical Group, Riyadh, Saudi Arabia; 3 Dr. Sulaiman Al Habib Medical Group, Alkhobar, Saudi Arabia; 4 Department of Nursing, Arabian Gulf University, Manama, Bahrain; 5 Medical and Surgical Nursing Department, Faculty of Nursing, University of Tabuk, Tabuk, Saudi Arabia; 6 Department of Community Health Nursing, College of Nursing, Jouf University, Sakakah, Saudi Arabia

**Keywords:** artificial intelligence, computer vision, external validation, health equity, machine learning, rehabilitation

## Abstract

**Background:**

Rehabilitation faces a scale problem: millions who could benefit lack timely, effective services. Artificial intelligence (AI) and device-based modalities (e.g., robotics and VR) can extend reach and personalise care when validated, yet decision-makers lack a consolidated view of clinical usefulness, translation to practice, safety, equity, and cost.

**Methods:**

We conducted an umbrella review of reviews using a Population–Exposure–Outcome framework. Searches span biomedical, allied health, and engineering databases from inception to September 1, 2025. We distinguished AI-enabled (ML/DL) interventions from technology-assisted (no ML demonstrated) modalities and synthesised outcomes across impairment, activity, independence, usability/safety, equity, and economics.

**Findings:**

The most reproducible clinical signal is activity improvement for post-stroke upper limb with technology-assisted training (robotics with or without VR) that increases task-specific practice; effects on impairment and independence are inconsistent once dose is matched and assessors are blinded. Claims of non-inferiority are not established when prespecified margins and confidence-interval testing are absent, so parity is interpreted as no between-group advantage under those conditions. Across AI-enabled domains, a development-to-deployment performance drop is evident most notably for brain–computer-interface classifiers and computer-vision movement evaluation limiting immediate clinical impact. Imaging-based decision support (radiomics/CNN) is closer to practice but varies by software and site, requiring local calibration and impact evaluation before pathway change. Reported adverse events are generally mild, yet usability, adherence, equity, and cost are under-measured, particularly in home and hybrid delivery. Prediction-model and trial reporting frequently fall short of contemporary AI standards; representation skews toward high-income settings, and subgroup performance is seldom reported.

**Conclusion:**

An adjunct-first posture is warranted. Adoption should be gated by minimum clinically important difference–anchored benefit under dose symmetry and blinded assessment; external, multi-site validation with declared lab-to-clinic performance loss; subgroup fairness with mitigation; decision-grade economic value; interoperability; and readiness for regulation, change control, and cybersecurity. Priorities include pragmatic, multi-site, assessor-blinded, dose-matched trials; standardised safety/usability capture for home use; and a public, living evidence atlas. AI can expand rehabilitation when held to clinical standards that matter to patients and services. With clear adoption gates and continuous post-market monitoring, systems can extend access and independence without sacrificing rigour, safety, equity, or fairness.

## Introduction

Rehabilitation is confronting a scale problem: an estimated 2.41 billion people are living with conditions that would benefit from rehabilitation, and the global need has risen by about 63% since 1990 as populations age and chronic diseases expand (1).

This persistent demand–capacity gap has intensified interest in artificial intelligence (AI) to augment delivery across the rehabilitation continuum, from acute care through community and home settings (2).

AI-enabled approaches now span brain-computer interfaces (BCIs) that decode motor intent to drive task practice, computer-vision and wearable-sensor models that grade movement quality and dose in real time, and predictive analytics that estimate functional trajectories to inform earlier triage and individualized planning (3–5).

Despite rapid growth, the literature on “AI in rehabilitation” is sprawling and siloed by modality, condition, and setting, making it difficult for clinicians and policymakers to see the whole field (6).

Several cross-cutting uncertainties justify a scoping umbrella review of reviews focused on clinical usefulness rather than technology performance alone. First, generalizability remains a central risk: models that perform well in single-site or development datasets often degrade when transported across hospitals, scanners, home environments, or patient populations, underscoring the need for external validation, local calibration, and post-deployment monitoring (7). Contemporary evaluations in clinical AI document performance decay under data shift and advocate operational monitoring frameworks to sustain accuracy and equity, concerns that directly affect vision-, sensor-, and prediction-driven rehabilitation tools (8).

Second, interpreting apparent “AI benefits” requires attention to exposure and assessor blinding (9). Third, safety and usability reporting particularly for home and hybrid pathways where AI could extend reach the most remains inconsistent, even as pooled data suggest low rates of mostly mild adverse events during telerehabilitation (≈0.3% of 84,534 sessions), arguing for standardized safety practices, explicit “hold” criteria, and structured adverse-event capture as AI shifts more dose into unsupervised environments (10).

Thirdly, translation pressure is increasing as AI systems move from pilot to practice, yet regulatory milestones or single-center successes do not establish clinical usefulness across diverse populations and settings, reinforcing the need for a joined-up picture of effectiveness, validation rigor, safety, usability, and equity (11–13).

Throughout this umbrella review, ‘rehabilitation’ refers primarily to adult neuro- and musculoskeletal rehabilitation (e.g., post-stroke upper-limb recovery, non-specific low-back pain, musculoskeletal physiotherapy) delivered in hospital, outpatient, community or home settings. We also include select evidence from paediatric conditions (e.g., cerebral palsy, paediatric participation) and speech/language rehabilitation where AI systems have been deployed. The review spans all adult age groups and includes some paediatric populations when relevant.

Given the rapidly expanding and heterogeneous literature encompassing diverse AI modalities, conditions and settings, no single systematic review can capture the entire field. Umbrella reviews are high-level syntheses of existing systematic reviews and meta-analyses that provide user-friendly summaries of the evidence base and allow clinicians and policy-makers to quickly understand what multiple syntheses have found. By collating and comparing systematic reviews, umbrella reviews can identify consistencies, discrepancies and gaps across interventions, populations and outcomes. This approach is particularly valuable when evidence is broad and fragmented, as it enables the development of common frameworks and informed guidelines for AI-enabled rehabilitation.

## Aim

This review aims to provide an integrated map of artificial-intelligence–enabled rehabilitation, aligning observed effects to the outcome levels namely impairment, activity, and participation and to translate these findings into practice-ready guidance for clinicians and policy makers.

### Objectives

- To determine across rehabilitation conditions where artificial-intelligence–enabled interventions yield clinically meaningful improvements at the levels of impairment, activity, and participation, and where apparent gains disappear under matched training dose and blinded assessment.
- To quantify the gap between development accuracy and real-world clinical performance, and to appraise validation rigour for brain–computer interfaces, vision-based movement evaluation, and imaging-based prognostic tools, including subject-wise, site-wise, cross-view, and external evaluation.
- To characterise present-day deployment readiness by synthesising evidence on safety, usability, adherence, home feasibility, regulatory clearance, and equity of representation, and to identify the immediate evaluation priorities

### Definitions

- **AI-enabled:** interventions that implement machine-learning or deep-learning algorithms which adapt, personalise or predict therapy.
- **AI-assisted:** interventions where AI is present but does not adjust in real time (e.g., automated reminders).
- **Technology-assisted:** robotics, virtual reality or other devices without a machine-learning component.
- **ΔReal:** the pre-specified difference between development (lab) performance and real-world performance.
- **MCID:** minimal clinically important difference – the smallest change in an outcome that patients perceive as beneficial.

## Methods

### Design

**Figure 1:**
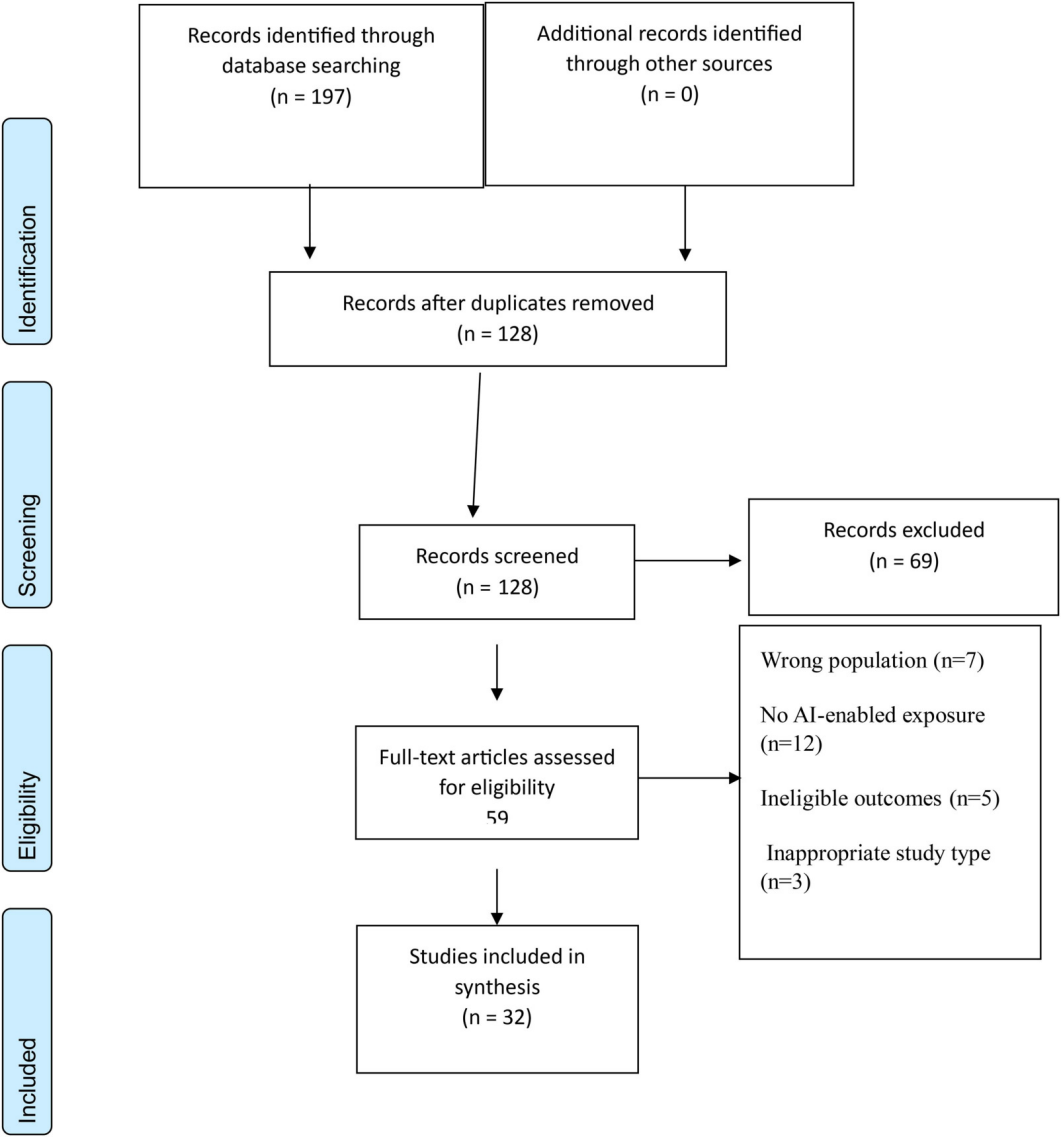
PRISMA 2020.

We conducted an umbrella review of reviews to map and critically interpret decision-relevant evidence on artificial-intelligence–enabled rehabilitation across modalities, conditions, and settings. We also followed the Preferred Reporting Items for Systematic Reviews and Meta-Analyses – PRISMA 2020 to ensure appropriate framing and reporting of a synthesis (Figure 1) (14). Because this is an overview of reviews, we aligned structure and terminology with the Preferred Reporting Items for Overviews of Reviews guidance to minimise redundancy and clarify how we handled overlap across included reviews (15).

We structured eligibility using Population–Exposure–Outcome (rather than a single-intervention PICO), because our analytic focus is the exposure “artificial-intelligence–enabled rehabilitation” as deployed in heterogeneous service contexts, comparators, and device families an approach recommended for mapping broad, multi-modality literatures (16). In the umbrella reviews, formal study-level critical appraisal is optional and should be undertaken only when it directly informs the review's aims (14).

Our priori objective was to chart the breadth of clinical effectiveness, real-world performance, safety, usability, and equity signals across review-level syntheses and to translate these into practice-ready guidance, not to derive pooled effect estimates or make comparative efficacy claims that would hinge on weighted risk-of-bias judgments.

We define the real-world performance gap (ΔReal) as the difference between development accuracy and external, multi-site, patient-inclusive performance, reported with confidence intervals and a prespecified acceptability margin tied to clinical consequences. Consistent with Joanna Briggs Institute and allied guidance, we therefore did not perform formal methodological quality or risk-of-bias appraisal of the primary studies nested within the included reviews, because such appraisal is not mandatory for scoping objectives and risks implying a level of comparative inference that the heterogeneity and overlap in this literature do not support (17). Instead, we documented design features that influence interpretation (for example, assessor blinding, dose symmetry, external validation, equity reporting) and we used established methods to describe and manage overlap among reviews rather than to exclude on the basis of risk-of-bias scores (18).

This umbrella review synthesised systematic reviews, scoping reviews and conceptual papers to map the evidence on AI-enabled and technology-assisted rehabilitation. We did not perform a formal risk-of-bias assessment or GRADE the certainty of evidence. Statements such as “most reproducible clinical signal” or “no consistent superiority” in the Results and Discussion are descriptive summaries rather than formal judgments of certainty. Recommendations regarding adoption frameworks (e.g., LAIR-REHAB) are conceptual proposals derived from observed evidence gaps and should be interpreted accordingly.

### Sources and search

We searched MEDLINE, Embase, Web of Science, Scopus, CINAHL, and IEEE Xplore from database inception to 1September 2025, combining controlled vocabulary and free-text terms for artificial intelligence modalities (for example, machine learning, deep learning, computer vision, wearable sensors, brain–computer interface, language models) and rehabilitation contexts (for example, physical therapy, occupational therapy, neurorehabilitation, telerehabilitation). The full database-specific strategies are provided in Supplementary Table S1. We chose a broad, concept-driven strategy to capture evaluation research, deployment and implementation reports, and model-development papers explicitly linked to rehabilitation use (19).

### Screening and selection

All records were exported and screened in Rayyan to enable blinded title/abstract assessment, deduplication, and decision tracking; Rayyan's predictions were not used to determine eligibility (20). Full texts were assessed against the prespecified Population–Exposure–Outcome criteria (Table 1).

**Table 1:** Population–exposure–outcome framework (operational definitions).

Element	Operational definition	Examples used to judge eligibility
Population	People receiving rehabilitation assessment or therapy across hospital, community, or home settings	Post-stroke upper limb programmes; musculoskeletal exercise therapy; cognitive or language rehabilitation
Exposure	An artificial-intelligence–enabled component that adapts, personalises, predicts, or guides therapy or triage	Computer-vision exercise feedback; wearable-sensor models with real-time coaching; brain–computer interface with functional electrical stimulation; language-model–based self-management coaching; imaging-based prognostication used to plan rehabilitation
Outcome	Patient-important endpoints and deployment-relevant signals	Function, independence, participation, quality of life; adherence, exposure, usability; adverse events; external validation and calibration; equity by PROGRESS-Plus factors; cost and budget signals

### Data extraction

Using a piloted template, we extracted for each included review: bibliographic details; AI modality and system features (for example, algorithmic adaptation, sensing, feedback, human-in-the-loop); rehabilitation context and setting; review time window and coverage; number of included studies and, where reported, participants; primary outcomes and whether they mapped to impairment, activity, or participation; presence of minimum clinically important difference anchoring or responder analyses; safety and usability reporting; adherence and exposure capture; external validation and calibration (for model-centric reviews); subgroup performance and equity variables guided by PROGRESS-Plus; and any economic or implementation signals.

### Evidence synthesis

Given substantial heterogeneity in modalities, comparators, outcomes, and follow-up timing along with asymmetric telemetry between trial arms in many primary studies we used a thematic and configurative synthesis rather than meta-analysis, reporting in line with Synthesis Without Meta-analysis guidance for transparency of grouping, metrics, and presentation (21). Quantitative effect directions and contextual notes were tabulated where a review provided consistent contrasts; qualitative findings (for example, usability, equity, workflow, adoption barriers) were aggregated using thematic synthesis methods (coding of results, generation of descriptive themes, and development of analytic themes) to integrate cross-cutting implementation signals.

### Inclusion and exclusion criteria

Table 2 summarises the inclusion and exclusion criteria applied at title/abstract and full-text screening.

**Table 2:** Inclusion and exclusion criteria (umbrella review of reviews).

Domain	Inclusion	Exclusion
Population	Humans receiving rehabilitation or rehabilitation-led prevention in physiotherapy, occupational therapy, or neurorehabilitation	Non-rehabilitation indications; animal or *in-vitro* research
Exposure	Artificial-intelligence–enabled rehabilitation that recognises, adapts, personalises, or predicts (for example, computer vision guidance; wearable-sensor with machine-learning feedback; brain–computer interface; language-model coaching; imaging-based triage used to plan rehabilitation)	Pure telehealth or video without algorithmic adaptation; sensors or platforms with no artificial-intelligence component; general informatics not used in rehabilitation
Outcomes	Patient-important clinical outcomes (pain, function, disability, quality of life); independence and participation; adherence and engagement; usability and patient experience; artificial-intelligence-attributable adverse events; for model studies: discrimination, calibration, internal and external validation, subgroup performance, and decision-curve or impact evidence	No eligible outcomes reported
Study type	Reviews that synthesise primary studies (for example, systematic reviews, meta-analyses, scoping reviews, narrative reviews with systematic methods); and review-level evidence that integrates model development and validation in rehabilitation use	Protocols, editorials, comments, letters without synthesis; unavailable full text
Language	English	Non-English
Time window	Inception to 1 September 2025	After 1 September 2025

In this review, we distinguish between AI-enabled interventions that incorporate adaptive or machine-learning algorithms and technology-assisted interventions such as non-adaptive robots or virtual-reality systems that provide fixed or pre-programmed feedback. We catalogued primary-study overlaps across reviews and described redundancy using the corrected covered area (CCA) where appropriate to avoid double-counting influence in our narrative conclusions. Where reviews addressing similar questions reached discordant conclusions, we used the Jadad decision algorithm as a diagnostic framework to explain differences (for example, eligibility, outcomes, analysis), not as a tiebreaker to exclude evidence.

### Data management and reproducibility

All screening decisions and data-extraction fields were logged in Rayyan with full audit trails to support reproducibility (20). We report our grouping decisions, synthesis logic, and presentation choices in line with synthesis without meta-analysis to maximise transparency in the absence of quantitative pooling (22, 23).

#### Intervention classification

We prospectively classified each review as AI-enabled (machine-learning–based), technology-assisted (no machine learning demonstrated), mixed (AI plus technology-assisted), or unclear. An intervention was considered AI-enabled only when machine- or deep-learning algorithms were both implemented and explicitly reported. Device-only modalities such as robotics, VR/AR, BCI, wearables, or app-based systems without an implemented ML/DL component were classified as technology-assisted. Where a review or primary study spanned both categories, we labelled it mixed. Throughout, we did not attribute effects from robotics/VR/BCI to “AI” unless ML/DL control, decision, or evaluation models were demonstrably incorporated and reported.

#### Review overlap

To quantify overlap between reviews, we extracted each review's list of primary studies and calculated pairwise CCA following Pieper et al. (2014). We interpreted CCA values as slight (0%–5%), moderate (6%–10%), high (11%–15%), and very high (>15%).

## Thematic synthesis

### Characteristics of included studies

We included 32 reviews (24–55) spanning stroke, musculoskeletal and spine disorders, cerebral palsy and paediatric participation, aphasia, myasthenia gravis, neuroregeneration, and cross-cutting rehabilitation practice. By design, 14 were systematic reviews (including three pairwise meta-analyses and one network meta-analysis), five were scoping reviews, and 13 were narrative, perspective, bibliometric or concept pieces. Bibliometric work mapped the field-level AI literature, whereas concept and perspective papers outlined frameworks and adoption considerations rather than pooled clinical effects.

To avoid over-attributing device effects to “AI,” we classified each review by the interventions actually studied. Eight reviews evaluated implemented machine-learning or deep-learning systems such as computer-vision pose or skeleton analysis, radiomics/CNN imaging support, brain–computer-interface classifiers, prediction/decision models, and NLP/chatbots and are labelled AI-enabled (ML-based). Four reviews examined robotics/VR/app/wearable interventions with no implemented ML/DL and are labelled technology-assisted (no ML demonstrated). Nineteen covered a mixture of algorithmic systems and device-only modalities (e.g., robotics with or without AI control, VR platforms alongside ML analytics) and are labelled mixed. One concept paper discussed AI potential without implemented models and is labelled unclear/narrative (no ML demonstrated). These tallies match Supplementary Table S2, for each review, where content is AI-enabled vs. technology-assisted.

Populations were dominated by post-stroke rehabilitation upper-limb motor outcomes, imaging-based prognosis, and BCI-assisted control with additional coverage of low-back pain, spine, cerebral palsy, aphasia, myasthenia gravis, neuroregeneration, and broad physiotherapy. Delivery settings included inpatient rehabilitation facilities, outpatient clinics, and home or tele-rehabilitation. Several reviews foregrounded home or hybrid models, including virtual rehabilitation, app-based dosing, and wearables or IMUs for adherence and monitoring. Many AI-enabled evaluations were lab-centric (e.g., pose/skeleton datasets and BCI classifier benchmarks), whereas the robotics and exoskeleton literature frequently reported prototype or pre-clinical readiness.

Outcomes were harmonised to impairment, activity, and independence (e.g., FMA-UE, ARAT, MBI) for clinical trials of technology-assisted modalities, and to algorithmic performance metrics for AI-enabled systems (e.g., AUC, accuracy, MAE/MAD/RMSE, and information-transfer rate for BCI). Imaging-support reviews reported discrimination and calibration statistics (e.g., mRS or infarct prediction) and emphasised site or software variability and the need for local calibration. For telerehabilitation and home use, usability, adherence, and safety were inconsistently reported and seldom standardised.

Across device-trial syntheses, the most reproducible signal is activity improvement for post-stroke upper limb with robotic training with or without VR that increases task-specific practice; effects on impairment and independence are inconsistent when dose is matched and assessors are blinded. Meta-analyses in non-specific low-back pain did not show added benefit over usual physiotherapy for pain or function. The network meta-analysis of technology-assisted modalities (robotics/VR/BCI) yielded probabilistic SUCRA rankings but limited pairwise superiority; accordingly, these device effects are not attributed to “AI” in the absence of implemented ML/DL. Across AI-enabled domains, we observed a consistent development-to-deployment performance drop for example, in BCI classifiers and computer-vision movement evaluation which limits immediate clinical impact without external, multi-site validation and impact evaluation. Imaging-support systems appeared closer to practice than other AI areas but varied by vendor, software, and site; local calibration and pathway-level evaluation are prerequisites to adoption. Risk of bias in systematic reviews of device trials was variable, with blinding and dose symmetry as recurrent constraints. We did not accept non-inferiority claims where prespecified margins and confidence-interval testing were absent; in such cases, parity is interpreted as no between-group advantage under study conditions. AI-enabled reviews frequently reported incomplete model transparency (limited availability of code or models and under-reporting of calibration, costs, or explainability). Representation was skewed toward high-income settings, subgroup performance was seldom reported, and fairness or equity analyses were uncommon. Adverse events were generally mild, but usability, adherence, and cost were under-measured, especially in home and hybrid delivery.

Only a subset of reviews provided pooled participant counts. The network meta-analysis of post-stroke upper-limb interventions included 4,702 participants across 101 RCTs; a pairwise meta-analysis of post-stroke robotics included ∼213 participants (seven RCTs; six pooled for BI/MI); and low-back-pain trials pooled ∼2,147 participants (eight studies; six meta-analysed). Most AI-enabled reviews did not aggregate participants, instead synthesising algorithm performance across datasets or reporting device or platform feasibility.

To reduce double counting, we mapped primary-study overlap via a citation matrix and, where feasible, characterised overlap using CCA. We retained all eligible reviews because observed overlap was modest and below common thresholds for “very high” redundancy; in the narrative synthesis we avoided double counting by attributing effect signals to their primary sources and down-weighting conclusions where overlap inflated counts.

From our thematic synthesis we identified four cross-cutting themes: (1) clinical effectiveness by outcome domain and modality; (2) the real-world performance gap and validation rigor; (3) technology effect vs. dose and engagement; and (4) deployment readiness, safety/usability, equity, and near-term priorities. Table 3 summarises the four major themes identified in our thematic synthesis. For each theme, we list the key findings, provide representative quantitative metrics (where available).

**Table 3:** Summary of key themes.

Theme	Key Findings	Representative Quantitative Metrics (with citations)	Notes
1. Clinical effectiveness by outcome and modality	Activity-level improvements are most consistent for post-stroke upper-limb robotics/VR; impairment and ADL improvements are inconsistent.	Post-stroke upper-limb robotics vs. conventional therapy: SMD 0.14 (95% CI 0.02–0.26) upper-limb capacity; no significant effect on ADL (SMD 0.04, 95% CI −0.05–0.13).	Minimum clinically important differences often not reported; benefits may reflect increased practice dose rather than AI effect.
2. Real-world performance gap and validation rigor	AI models/devices often perform worse in real-world settings than in lab.	Brain–computer interfaces: offline classification accuracy of ∼98.8% vs. online accuracy of ∼50%; other BCI studies show offline accuracy 64.5–97.0% vs. online 50–83%, representing a 20–50% drop.	Many computer-vision and sensor models lack external validation; subject-wise or cross-site performance drops are seldom quantified.
3. Technology effects vs. dose and engagement	Benefits often diminish when training dose is matched and assessors are blinded.	Low-back pain: no significant advantage of AI-assisted physiotherapy over usual care for pain/function; parity on ADL outcomes.	Reporting of repetitions, minutes on task, and adherence is inconsistent, limiting mediation analyses.
4. Deployment readiness, safety, equity	Safety events are generally mild; usability, adherence, equity and cost are under-measured.	Few quantitative metrics reported; adverse events mainly minor (e.g., skin irritation, fatigue) and rare in tele-rehab (∼0.3% of sessions).	Descriptive; emphasise under-reporting of usability, adherence, equity, and economics.

SMD, standardised mean difference; ADL, activities of daily living; BCI, brain–computer interface; ML,  machine learning; VR,  virtual reality; DL,  deep learning.

### Theme 1: clinical effectiveness by outcome domain and modality

We group outcomes into three domains: impairment (body structure and function), activity (task performance), and independence in daily activities. For orientation, the Fugl–Meyer Assessment of the Upper Extremity represents impairment, the Action Research Arm Test represents activity, and the Modified Barthel Index represents independence. Across the reviews synthesised here, minimum clinically important differences, responder analyses, adverse events, adherence, and cost-effectiveness are rarely reported or pooled. Unless explicitly stated, “technology-assisted” refers to robotics, virtual reality, brain–computer interface, and related systems; many included trials used automation or fixed or adaptive control without a demonstrated machine-learning component, so “artificial intelligence” should not be inferred universally.

#### Subtheme 1.1: activity gains concentrate in robot training with or without virtual reality for post-stroke upper limb

A large, bilingual network meta-analysis of randomised trials found that technology-assisted interventions improved Action Research Arm Test scores vs. conventional therapy, with robot training combined with virtual reality among the highest ranked on this activity measure (44). In contrast, impairment-level outcomes (total and distal Fugl–Meyer Assessment of the Upper Extremity) and independence (Modified Barthel Index) showed no significant between-group differences. The surface under the cumulative ranking curve conveys ordering probability under model assumptions and does not establish clinical superiority. Interpretation is further constrained by incomplete loops in several networks (limiting inconsistency checks), heterogeneous devices and comparators, mixed stroke phases and severities, and likely exposure and repetition imbalances favouring intervention arms. Method-focused reviews concur that technology-assisted can deliver task-specific benefits in reaching, grasping, and manipulation, but effects are context-dependent across device classes and settings and often attenuate when outcome assessors are blinded and exposure is matched (38, 39). Safety and adherence are under-reported.

#### Subtheme 1.2: impairment and daily-independence effects are inconsistent once exposure is matched

A meta-analysis restricted to randomised trials in stroke reported no between-group superiority on the Barthel Index or Modified Barthel Index, and an imprecise, heterogeneous estimate on the Motricity Index with high statistical heterogeneity (42). Other reviews note that apparent advantages often shrink when exposure is closely matched and outcomes are assessor-blinded (39, 45). Although the stroke meta-analysis discusses non-inferiority conceptually, no prespecified margin was tested against between-group differences with confidence intervals; non-inferiority is therefore not established. Overall, superiority on impairment and on independence remain unproven and is sensitive to risk of bias, heterogeneity, and dosing. Notably, the implementation-focused review reported that no intervention had regulatory certification at the time of its search (45), underscoring translational limits.

#### Subtheme 1.3: outside post-stroke upper limb, pooled effects are small or uncertain and rarely anchored to minimum clinically important difference

Findings beyond post-stroke upper limb are domain- and condition-specific. In non-specific low back pain, a randomised-trial meta-analysis found no significant pooled advantages of technology-assisted physiotherapy over usual physiotherapy for pain, function, or mental health; effects were small and directionally inconsistent (43). One table shows a confidence interval that appears to exclude zero alongside a non-significant probability value; this likely reflects a rounding or reporting error and does not change the overall “no clear benefit” conclusion. Scoping and narrative reviews across physiotherapy and technology-assisted rehabilitation consistently highlight sparse reporting of minimum clinically important differences and responder proportions, heterogeneous end points, limited assessor blinding, and under-synthesis of harms, adherence, and costs (36, 37, 55). These observations should not be generalised to every non-stroke indication without dedicated syntheses in those conditions. The LAIR-REHAB 2.0 domains and minimum standards are summarised in Table 4.

**Table 4:** LAIR-REHAB 2.0: core domains, minimum standard, proof-of-compliance.

Domain	Tiered minimum (pass-check)	Proof of compliance (examples)
Outcomes & MCIDs	T1: prespecified ICF-mapped outcome+blinded where feasible; T2: MCID/responder analysis under dose symmetry+blinded assessors; T3: sustained benefit & responder heterogeneity	SPIRIT-AI/CONSORT-AI excerpts; SAP; MCID sources; blinded-assessor log
Dose & exposure	T1: transparent dose/telemetry; T2: dose symmetry (reps/min/contact) across arms; T3: exposure distributions & adherence by subgroup	Telemetry schema; raw exposure histograms; adherence curves
ΔReal (generalisation)	T1: internal vs. subject-wise (or cross-view) gap; T2: multi-site external validation with CI and margin; T3: home-use validation & drift monitoring	ΔReal sheet (definitions, numerators/denominators, CIs); multi-site report; drift triggers
External validity	T2+: calibration (intercept/slope), subgroup performance, decision curves	Calibration plots; subgroup tables; net-benefit curves; PROBAST-AI summary
Behavioural safety	T1: automation-bias watchlist; T2: pre-specified AI-off audits; T3: remediation & re-audit	Unaided-performance trajectories; mitigation protocol; re-audit results
Safety & usability	T1: AE taxonomy+failure logs; T2: hold/escalation rules; T3: remote-support SOPs & validated usability	AE CRFs; failure-log extracts; hold criteria; SUS/UEQ scores; support SOP
Equity & access	T1: dataset card; T2: parity thresholds/bounds+uptake/adherence by subgroup; T3: low-bandwidth, multilingual, accessibility plan & device/connectivity provisioning	Dataset card; equity impact statement; subgroup uptake/adherence; provisioning logs
Regulation & security	T2: pathway mapping (e.g., EU AI Act class); T3: FDA PCCP/learning bounds, SBOM, threat model, vulnerability disclosure & patch SLA	Classification memo; PCCP doc; SBOM; threat model; CVDP; SLA
Site calibration (imaging)	T3 (if imaging used): local agreement, protocol dependencies, drift monitoring	Local calibration study; acceptability thresholds; drift logs
Economics	T2: CHEERS-2022 core items; T3: CHEERS-AI items, ICER/QALY, budget impact, sensitivity	CHEERS checklist; CHEERS-AI; model files; payer scenario analyses
Interoperability & model card	T2: HL7 FHIR resources (or roadmap)+model card; T3: SMART-on-FHIR launch/scopes, TEFCA alignment, post-market plan/registry	FHIR conformance; API catalogue; model-card URL; post-market plan; registry entry

Technology-assisted training, especially robotics with or without virtual reality shows consistent activity-level gains in post-stroke upper limb, while impairment and independence advantages are not convincingly superior to well-delivered conventional therapy once exposure and bias are controlled. Across conditions, clinical meaningfulness is hard to judge because minimum clinically important differences, responder proportions, safety, adherence, and cost-effectiveness are rarely synthesised.

### Theme 2: real-world performance gap and validation rigor

Across the reviews synthesised here, models and devices that look strong in laboratory settings or convenience samples often perform materially worse in real clinical use (35, 39, 55). This gap limits confidence in laboratory-reported accuracy and argues for site-specific external validation and calibration tied to patient-important outcomes functional use, independence, participation, safety, adherence, and quality of life rather than technical proxies (34).

#### Subtheme 2.1: brain–computer interfaces drop from offline to online use and from healthy volunteers to patients

Visual-evoked-potential brain–computer interfaces for motor rehabilitation are still evaluated mainly in offline analyses with healthy volunteers. Only a small fraction of studies includes patients, and where online control in patients is reported, both accuracy and information transfer rate typically fall relative to offline analyses in healthy cohorts (33). Broader neurorehabilitation reviews echo the scarcity of patient-inclusive online trials and recommend shifting from accuracy or information-transfer proxies toward clinically salient endpoints (functional use, independence, participation) with safety and adherence measured alongside (34, 52). Early regulatory entries such as an electroencephalography-driven hand orthosis described in the United States Food and Drug Administration review underscore promise and the need for prospective, site-specific evaluation before routine adoption, since available evidence is small and often single-arm (32).

#### Subtheme 2.2: skeleton- and vision-based exercise evaluation degrades under rigorous validation and in real settings

Systematic assessments emphasise small, lab-centred datasets, single-view capture, and heterogeneous metrics. Performance commonly falls under participant-wise or cross-view validation compared with frame- or sequence-wise splits, signalling limited generalisation from curated laboratories to homes and clinics (35, 39). Head-to-head benchmarks show that topology-preserving, spatio-temporal models outperform hand-engineered pipelines on curated sets, yet absolute errors increase as evaluation rigour rises (39). Recommended remedies include richer, multi-view, clinician-scored datasets (several widely used public datasets in this area rely on non-clinician labels), standardised splits and metrics, external validation, and per-segment or per-joint feedback anchored to clinical scales and minimum clinically important differences (35, 39).

#### Subtheme 2.3: imaging-based artificial intelligence is deployable but software- and site-sensitive; outcome-relevant use needs local calibration

Technologies cleared by the United States Food and Drug Administration for computed tomography, computed tomography angiography, and computed tomography perfusion tasks often match expert performance on specific targets and reduce time to notification. However, infarct-core and penumbra estimation and automated Alberta Stroke Program Early Computed Tomography Score vary by software package and protocol, with larger errors in very large cores or extreme hypoperfusion and with differences across scanners and sites (32). Direct evidence that imaging-based predictions improve rehabilitation outcomes remains limited; use should be considered hypothesis-generating until impact is demonstrated locally. If outputs are to influence rehabilitation timing, therapy intensity, or discharge planning, services should require local calibration and prospective evaluation of clinical impact, along with public model documentation, site-specific external validation, and post-deployment monitoring for drift and bias; avoid hard thresholds for rehabilitation allocation without demonstrated local utility (34, 56).

#### Subtheme 2.4: treatment dose vs. technology in trials: parity is common

Across rigorous trials and syntheses, technology-assisted rehabilitation (with or without artificial-intelligence components) often performs on par with dose-matched conventional therapy, reinforcing the laboratory-to-clinic performance gap. Pooled stroke randomised controlled trials show no superiority on functional independence measured by the Barthel Index and imprecise, heterogeneous effects on motor strength measured by the Motricity Index (42). Randomised trials in non-specific low back pain show no significant advantage for technology-assisted physiotherapy on pain, function, or mental health (43). A network meta-analysis across upper-limb stroke modalities finds activity-level gains on the Action Research Arm Test but no consistent advantage for independence in daily activities (44). Systematic review of clinically implemented AI found mixed, context-specific effects with no consistent superiority over standard care (45).

#### Subtheme 2.5: validation transparency and openness are the bottleneck

In physical therapy ML studies, subject-wise cross-validation, external testing, calibration/uncertainty reporting, and open artifacts (code/models/data) are frequently missing; only a small minority make models or datasets public, and costs/explainability are often under-reported (55). Vision/skeleton reviews similarly flag non-uniform metrics/splits and tiny, lab-centric datasets that hinder reproducibility and clinical translation (35, 39).

Headline accuracies from small, homogeneous, laboratory datasets tend to overstate clinically useful performance. To bridge laboratory promise to reliable, equitable benefits in routine care, prioritise participant-wise and cross-site validation, external testing, dose-matched designs, and pre-specified clinical endpoints (functional use, independence, participation, safety, adherence, cost-effectiveness, minimum clinically important differences, and responder proportions), with post-deployment monitoring for drift and bias and local calibration before models or devices inform real-world rehabilitation decisions (31, 34, 55).

### Theme 3: technology effect vs. dose and engagement effect

#### Subtheme 3.1: non-superiority on global outcomes under dose-controlled or blinded conditions

When trials enforce assessor blinding and/or match treatment dose, between-group differences on impairment and on independence in daily activities generally attenuate. The most recent pooled randomised controlled trial evidence in stroke shows no statistically significant superiority on the Barthel Index/Modified Barthel Index, and only an imprecise, heterogeneous signal on the Motricity Index plausibly related to device class, stroke phase, and trial quality (42). Syntheses in non-specific low-back pain and upper-limb stroke likewise show parity with usual care on global outcomes (e.g., ADL), and where dose is controlled in individual trials, apparent advantages tend to shrink (43, 44). Reviews emphasise that advantages attributed to “artificial intelligence” can partly reflect comparator quality and higher exposure rather than technology-intrinsic effects (29, 38, 39, 45). In several device-focused syntheses, the term “artificial intelligence” also encompasses robotics or virtual reality with unspecified or minimal learning components (taxonomy drift), so claims should be interpreted cautiously (29, 36, 38, 39, 44).

#### Subtheme 3.2: engagement and behavioural dose are frequently higher in intervention arms but are poorly captured

Across deployments, technology-assisted arms often deliver more repetitions, longer time on task, may include added therapist contact, or provide more engaging interfaces; where exposure is reported, groups with higher exposure tend to achieve larger gains. However, trials inconsistently report repetitions, minutes, adherence, and fidelity, which precludes formal mediation analyses of the dose-to-effect pathway (30, 36, 45, 55). Platform-level reviews describe architecture-driven adherence features wearables, reminders, and conversational agents with promising but under-measured real-world impact (28, 34). In paediatrics, the evidence base is small and largely clinic-bound, limiting conclusions about engagement in home and school contexts where participation occurs (27).

#### Subtheme 3.3: where superiority appears, it concentrates in post-stroke upper-limb activity; impairment and independence remain inconsistent

In contrast, apparent superiority often shrinks or disappears once dose is controlled and assessors are blinded. Consistent advantages concentrate in activity-level performance (ARAT) for post-stroke upper limb, where several technology-assisted modalities outperform usual care and robot training with or without virtual reality frequently ranks highest; ranking probabilities alone do not establish clinical superiority (39, 44). By contrast, impairment-level outcomes on the Fugl–Meyer Assessment of the Upper Extremity (total or distal) and ADL on the Modified Barthel Index show inconsistent between-group differences (42, 44). Claims of non-inferiority remain premature without a prespecified margin and confidence-interval testing against that margin (42). Imaging and regulatory syntheses further note that site and software sensitivity and workflow gains do not automatically translate into superior patient-centred outcomes (31, 32).

Many observed advantages track with greater exposure/engagement rather than technology-intrinsic effects. Where superiority is observed, it is most consistent for activity-level tasks in post-stroke upper-limb function and less consistent for impairment and independence. Future trials should equalise dose, blind assessors, and report detailed exposure and fidelity so the technology's contribution can be disentangled from behavioural dose.

### Theme 4: deployment readiness, safety and usability, equity, and near-term priorities

#### Subtheme 4.1: what was already in clinical use and what evidence accompanied it

Across the included reviews, a United States Food and Drug Administration–centred landscape described twenty-two artificial intelligence or machine-learning technologies for stroke, mostly for diagnosis or triage, with consistent reports of shorter time to notification and performance at or near expert level. Two rehabilitation-relevant devices were repeatedly noted: an electroencephalography-based brain–computer interface hand orthosis with *de novo* marketing authorization in 2021, and a machine-learning-guided, extremely low-frequency electromagnetic neuromodulation system with Breakthrough Device designation but without marketing authorization. Both devices were associated with early signals for upper-limb recovery; however, assessor-blinded, dose-matched randomized controlled trials demonstrating superiority on the Action Research Arm Test, the Fugl–Meyer Assessment of the Upper Extremity, or independence in daily activities were not identified in the reviewed material (32). For imaging-based prognostic models, the reviews consistently reported variation in outputs by software package, protocol, scanner model, and site. Evidence supporting use in care-pathway decisions emphasized time and workflow measures; direct links to rehabilitation intensity, timing, or discharge disposition were uncommon (31, 34). Economic evaluations were rarely synthesized, limiting cost-benefit inference alongside clinical utility.

#### Subtheme 4.2: safety, usability, adherence, and exposure reporting

Device-assisted rehabilitation studies most often reported mild adverse events such as skin irritation from orthoses or electrodes, fatigue, and cybersickness. Standardized usability instruments and systematic capture of adherence and exposure were infrequently reported, particularly in home-based or tele-rehabilitation deployments. Several reviews highlighted incomplete documentation of repetitions, minutes on task, and fidelity, limiting inferences about risk–benefit and implementation feasibility (28, 34, 39, 45).

#### Subtheme 4.3: equity and condition-specific patterns; proposals for home musculoskeletal designs

In paediatric participation studies, interventions were predominantly delivered in person and centred on robots; personalisation was usually categorical rather than tailored to child and family goals, and reporting of race, ethnicity, and socioeconomic variables was rare, raising concerns about representativeness and carry-over to home and school contexts (27). Scientometric mapping showed concentration of publications in high-income settings and elite institutions, suggesting potential dataset dominance and standard-setting hegemony (26). For aphasia, the review concentrated on assessment and diagnosis; evidence for therapy and self-management, including work using large language models, was sparse and rarely anchored to patient-centred endpoints or safety guardrails (25). For cerebral palsy, early video-based screening systems were described as promising but were not prospectively linked to earlier intervention or to downstream gains in function and participation within the reviewed evidence (24). Within neuroregeneration, reports involving technology-assisted and stimulation were heterogeneous, with early-stage samples and non-uniform outcomes; external validation and standardized outcome sets were uncommon (29). For tele-rehabilitation platforms, sensor-led programmes with conversational guidance were described as feasible but constrained by privacy, governance, and access. Reviews proposed design elements for home-based musculoskeletal programmes, including clear safety and pause criteria with discomfort detection and escalation pathways, adaptive progression bounded by therapist-set limits with per-joint or per-segment movement-quality metrics, clinician dashboards displaying adherence, number of repetitions, minutes on task, range-of-motion targets, and compensation indices, and equity features such as low-bandwidth modes, multilingual and accessible interfaces, caregiver-load tracking, and privacy-preserving on-device or federated learning approaches (28, 34, 36, 39, 45, 55).

In myasthenia gravis, proposals for artificial-intelligence-enabled long-term safety monitoring and self-management lacked controlled effectiveness data within the reviewed evidence (38). A service-design blueprint for physiotherapy integration was presented as a concept without implemented outcomes and therefore did not contribute efficacy data (53).

## Discussion

Building on our thematic synthesis, four messages recur across the included reviews.

First, the most consistent clinical signal is post-stroke upper-limb activity gains measured by the Action Research Arm Test especially with technology-assisted with or without virtual reality yet ranking probabilities do not establish clinical superiority, and clinical meaningfulness remains uncertain because minimum clinically important differences and responder proportions are seldom reported (39, 44). Second, between-group gains in impairment measured by the Fugl–Meyer Assessment of the Upper Extremity and in independence measured by the Modified Barthel Index generally attenuate under assessor blinding and dose-matched designs, indicating that higher behavioural dose and engagement likely contribute to part of the observed advantage; formal mediation is rarely possible given inconsistent exposure and adherence reporting (39, 42, 45). Third, a laboratory-to-clinic performance gap persists: brain–computer interface systems and computer-vision or skeleton-based movement-evaluation systems often show accuracies in offline analyses with healthy volunteers that overstate performance during online, patient-inclusive use, while imaging systems deliver time and workflow efficiencies yet remain sensitive to site and software differences and lack direct evidence of improved rehabilitation outcomes, requiring local calibration and prospective evaluation before changing pathways (31, 33–35, 39). Fourth, safety reports are largely mild, but usability, adherence and exposure, equity, workforce, and economic outcomes remain under-reported, particularly for home-based or hybrid delivery (27, 28, 30, 34, 45). Importantly, several technology-assisted trials used automation or fixed or adaptive control without a demonstrated machine-learning component; not all observed effects should be attributed to artificial intelligence.

We explicitly state that none of the systematic reviews reported effect sizes stratified by sex, race or socio-economic status; therefore, fairness and equity recommendations in the LAIR-REHAB framework are prospective proposals rather than evidence-based conclusions. Our recommendations throughout are framed as guidance for future research and implementation rather than prescriptive rules.

These patterns point to concrete priorities for the field: conduct dose-matched, assessor-blinded trials anchored to minimum clinically important differences and responder outcomes; require participant-wise and site-wise validation with external testing; and institute post-deployment monitoring with equity by design and cost reporting as first-class outcomes. We outline these implications and near-term recommendations next.

### Post-stroke upper limb: activity gains without proven superiority on impairment/ADLs

Task performance improves, but superiority on impairment or independence is not yet proven under rigorous conditions.

The large network meta-analysis (101 RCTs) shows consistent Action Research Arm Test improvements for robotic therapy, brain–computer interface, virtual reality, “intelligent or remote” rehabilitation, and robotic therapy plus virtual reality compared with conventional therapy (standardized mean differences approximately zero point seven to zero point nine), whereas Fugl–Meyer Assessment for the Upper Extremity (total and distal) and Modified Barthel Index contrasts are typically non-significant; Surface Under the Cumulative Ranking orderings are probabilistic and should not be read as clinical superiority in the absence of significant pairwise contrasts and minimal clinically important difference anchoring (44). An RCT meta-analysis likewise finds no superiority for AI-assisted programmes on Barthel or Motricity Indices (42). Reviews emphasise implementation realities and heterogeneity that limit generalisability beyond this niche (39, 45). Illustrative trials in similar settings report short-term gains in Action Research Arm Test and Wolf Motor Function Test or range of motion under enriched exposure, but with asymmetric telemetry and limited follow-up (57, 58).

For upper-limb stroke rehabilitation, artificial-intelligence-enabled programmes especially robotic therapy with or without virtual reality are reasonable adjuncts to lift task performance; claims of superiority on impairment or independence require assessor-blinded, dose-matched randomized controlled trials and minimal clinically important difference or responder reporting (42, 44).

### Outside post-stroke upper limb: modest or uncertain effects

Effects outside post-stroke upper limb are modest, mixed, or uncertain; adopt as capacity extenders, not replacements.

A meta-analysis of randomized controlled trials in non-specific low-back pain finds no significant pooled benefit of artificial-intelligence-assisted physiotherapy for pain, function, or mental health compared with usual care (43). Reviews across mixed conditions echo inconsistent effects and implementation constraints (39, 45). Several trials in interactive telerehabilitation report domain-specific improvements under facilitated, intervention-only telemetry, whereas an assessor-blinded three-arm trial of an artificial-intelligence self-management application showed no primary between-group differences; a multicentre non-inferiority trial in post-stroke cognition suggests non-inferiority of a self-guided, artificial-intelligence-personalised programme to supervised therapy on the Mini-Mental State Examination with similar Modified Barthel Index and EuroQol five-dimension questionnaire gains (59–61).

Outside upper-limb stroke, capacity-expanding deployment under guardrails is justified; replacement of well-delivered therapist care is not (43, 45).

### Dose, engagement, and comparator quality drive apparent benefit

When dose and assessment are fair and blinded, the apparent edge of technology often fades. Across the included reviews, apparent superiority wanes when assessor blinding and dose-matching are enforced (39, 42, 45). Objective exposure is often measured only in the AI arm (device logs), while controls rely on recall/paper logs an asymmetry that inflates technology effects; reinforcement-learning deployments may improve within-arm adherence and service efficiency without proving superiority to well-delivered care; mood and stress suppress engagement in pragmatic use (57, 62, 63). Trials should pre-specify dose-matching, symmetric telemetry in all arms, blinded assessment, and mediation analyses linking repetitions or on-task time to outcomes; endpoints should be minimal-clinically-important-difference-anchored (39, 45).

### ΔReal: a defining translational gap

Laboratory accuracy does not automatically translate to clinic or home**.** Methods-focused reviews show systematic drops from offline to online and from healthy participants to patient performance in robotic or brain–computer-interface pipelines and skeleton-based evaluators; dataset flaws (small, single-view, laboratory-centric) and non-standard splits overstate accuracy (35, 39, 41). In computer-vision exercise scoring, near-ceiling accuracy degrades under subject-wise or cross-view splits, and prognostic models frequently lack external validation, calibration, and impact analyses (64, 65).

### Safety, usability, and human factors are under-measured

Harms appear uncommon, but the field under-measures adverse events, failures, and real usability. Included reviews report few and mild adverse events but infrequent adverse-event capture, absence of pre-specified hold criteria, limited failure logs, and inconsistent usability instruments especially in home or telerehabilitation (28, 39, 45).

Scoping and perspective reviews highlight cost/availability, interoperability, governance, and training gaps; paediatric participation-focused AI is overwhelmingly in-person technology-assisted with minimal remote delivery and almost no individuated, goal-anchored personalization (27, 30, 34). Facilitators (digital-literacy support; scheduled clinician touchpoints) and moderators (depression and stress) materially affect adherence (60, 63).

### What is deployment-ready now?

Stroke imaging artificial intelligence is closest to routine use; most other areas are implementation feasible as extenders under guardrails. Of the domains mapped by the included reviews, stroke imaging artificial intelligence is closest to routine use for triage and prognosis, with package- and site-sensitive variation that mandates local calibration and drift monitoring before outputs influence early rehabilitation planning or disposition (31, 32). Other areas (interactive home musculoskeletal rehabilitation; self-guided cognitive rehabilitation) are implementation-feasible but should be adopted as capacity extenders under guardrails, not replacements (42, 43, 45).

Within the included reviews, artificial intelligence most reliably increases practice intensity and improves activity-level performance in post-stroke upper-limb programmes (particularly robotic therapy with or without virtual reality), while superiority on impairment and independence is not established under dose-matched, assessor-blinded, symmetrically measured conditions (39, 42, 45). Scale-up should be gated by validation that is aware of the real-world performance gap, by human-factors and equity safeguards, and by proportionate procurement aligned to evidence maturity (30, 32, 34, 35, 41).

### LAIR-REHAB 2.0 conceptual framework: a unifying framework to close the evidence–translation gap in AI-enabled rehabilitation

A tiered, risk-proportional threshold should govern publication, payment, and adoption, with modality- and maturity-specific gates rather than a single universal bar. Rehabilitation demand remains vast, people living with conditions that would benefit from timely, effective rehabilitation, underscoring the stakes for any technology that claims to scale access or improve outcome (1). To date, the most reproducible clinical signal appears in post-stroke upper-limb programmes where task-specific practice is intensified via technology-assisted; across recent meta-analyses, activity-level gains are more consistent than impairment or independence gains, which often attenuate under dose-matched, assessor-blinded conditions (66–68). This pattern supports adjunctive use today and justifies caution on claims of superiority for impairment or ADL until MCID-anchored responder analyses and rigorous comparators are demonstrated, while remaining open to future evidence at higher cumulative doses and with more personalized adaptation (69).

Translation risks are non-trivial across modalities: computer-vision exercise scoring frequently reports optimistic performance on small, laboratory datasets and weak splits, with accuracy falling under subject-wise or cross-view validation that better reflects home capture. Brain–computer interfaces continue to show a discrepancy between offline accuracy in healthy participants and online control in patient cohorts, limiting immediate generalisability to durable functional benefit. Stroke triage/prognostic imaging is closer to practice, yet automated ASPECTS and perfusion outputs can vary by software and protocol, necessitating local calibration and drift monitoring before pathway changes that would alter the timing or intensity of rehabilitation. Operationalising generalisation explicitly via ΔReal is required, but phase-appropriate: early studies should report internal vs. external (or subject-wise) splits; subsequent phases should add multi-site and home validation with pre-specified non-inferiority margins. Emerging data in specific domains (e.g., gastrointestinal endoscopy) show a behavioural-safety hazard consistent with automation bias/deskilling; clinician-assistive workflows should monitor and mitigate this via scheduled “AI-off” periods and skills refresh, without obscuring net benefits when AI is on.

LAIR-REHAB 2.0 operationalises these realities into auditable requirements that align with contemporary reporting, regulation, and equity standards. For studies that develop or evaluate prognostic/triage models, compliance with TRIPOD-AI and PROBAST-AI is mandatory where applicable (with a comply-or-explain provision for edge cases) to specify models, handle bias, and report subgroup performance transparently (70, 71). Early, live clinical evaluations of AI-enabled interventions should follow DECIDE-AI to capture workflow fit, usability, and failure modes before definitive trials (72). Protocols and trial reports must implement SPIRIT-AI and CONSORT-AI extensions so that primary outcomes, allocation, and AI–human interaction are prespecified and reproducible (73, 74). Economic claims should meet CHEERS-2022 while using CHEERS-AI to disclose AI-specific cost drivers (learning over time, autonomy, monitoring); economic evaluations are stage-appropriate (recommended for pivotal/late-phase studies and for reimbursement dossiers, not necessarily for small pilots) (75, 76).

Regulatory-security readiness is no longer optional. The World Health Organization's guidance on large multimodal models adds further guardrails on oversight triggers, privacy, and risk mitigation for chatbot and coaching components that increasingly sit atop sensor-first telerehabilitation (77).

Equity-by-design must be evidenced rather than asserted. Transparent dataset cards that quantify representativeness and missingness, with predefined subgroup-parity thresholds (or justified bounds) on performance and low-bandwidth, multilingual operation, are baseline expectations under emerging dataset transparency guidance and should be auditable in both publications and product dossiers. When parity gaps are identified, mitigation plans, and re-audit timelines should be reported.

LAIR-REHAB 2.0 sets tiered, enforceable thresholds for journals, payers, and health systems (Table 3):

- Tier 1 (Pilot/Feasibility): blinded primary outcomes where feasible; dose reporting; ΔReal (internal vs. subject-wise) disclosure; usability/failure logs.
- Tier 2 (Pivotal/Definitive): MCID-anchored clinical benefit under assessor blinding and dose symmetry; ΔReal with external/multi-site validation; equity analyses; stage-appropriate economics.
- Tier 3 (Deployment/Payment): documented equity and economics; regulatory, security, and interoperability readiness; site-calibration (imaging); post-market learning bounds and drift monitoring.

This tiering preserves rigor while avoiding an all-or-nothing gate that could stifle early-stage innovation.

LAIR-REHAB 2.0 removes one-size-fits-all thresholds and replaces them with modality-specific, outcome-anchored ΔReal metrics that make generalisation claims falsifiable in routine settings. It binds every study and product dossier to named standards TRIPOD-AI/PROBAST-AI, DECIDE-AI, CONSORT-AI/SPIRIT-AI, CHEERS-2022/CHEERS-AI, so reporting and appraisal become uniform and auditable. It embeds statutory obligations (EU AI Act) and deployment prerequisites (FHIR/SMART-on-FHIR/TEFCA) to shorten the distance from study to safe service change. It elevates behavioural safety to co-primary status in clinician-assistive scenarios in light of new deskilling evidence, preventing over-reliance harms in rehabilitation services. And it converts equity from aspiration to obligation via dataset cards, subgroup parity bounds, and low-resource operation as reportable artefacts.

### Implications and recommendations

Artificial-intelligence-enabled rehabilitation should be judged by the same standard we expect of any therapy: meaningful gains in function, independence, and quality of life for people who need rehabilitation, numbering roughly 2.4 billion worldwide, delivered safely, equitably, and at scale. The promise is real: technology-assisted, sensor-first telerehabilitation, brain–computer interfaces, and large-language-model coaching can raise practice intensity and personalise exercise. Yet the evidence is still maturing. The most consistent clinical signal appears in post-stroke upper-limb programmes, where robot-assisted and virtual-reality-assisted training increases task-specific practice and improves activity-level outcomes. By contrast, superiority on impairment and independence often diminishes when exposure is matched and assessors are blinded, implying that dose and engagement rather than the technology itself account for much of the observed benefit. In practice, artificial intelligence should augment, not replace, well-delivered therapy.

Future evaluations must make patient-important outcomes non-negotiable. Primary endpoints should be mapped to the International Classification of Functioning, Disability and Health across impairment, activity, and participation; anchored to minimal clinically important differences; and paired with pre-specified responder definitions. Trials should use assessor blinding, dose-matched comparators, and standardised protocols and timepoints. Because many artificial-intelligence systems work at least in part by increasing practice, analyses should incorporate mediation models that test whether extra repetitions or time on task explain outcome differences. Surrogate metrics such as device usage, movement smoothness, or algorithmic accuracy are not sufficient unless they are explicitly linked to real-world functional gains.

Translation to routine care is where most systems stumble, so the real-world performance gap must be measured and disclosed. A simple, modality-specific real-world performance gap should be specified *a priori* and reported with confidence intervals and an acceptability margin tied to clinical consequences. For computer-vision exercise evaluation, that means reporting external, subject-wise performance ideally on home-captured video against internal or within-subject baselines, and showing robustness across views, lighting, and devices. For brain–computer-interface systems, it means quantifying the paired drops from offline to online control and from healthy volunteers to patient cohorts, alongside information-transfer rates in the intended therapy setting. For imaging tools that shape triage or early rehabilitation planning, it means demonstrating site-specific calibration and disclosing package-to-package variance before any pathway change. Claims of superiority or non-inferiority should be contingent on these translational parameters, not on laboratory accuracy alone.

Safety, usability, and human factors require the same rigour as efficacy. Rehabilitation often occurs in homes and hybrid settings with limited direct supervision, so studies and products should define an adverse-event taxonomy that covers falls, fatigue, skin issues, and cybersickness; implement automatic error and failure logging; and include explicit hold and escalation criteria that pause unsupervised sessions when concerning patterns arise. Usability and adherence should be co-primary outcomes for home and community programmes, using validated instruments and documented remote support procedures. Because reliance on assistive automation can erode unaided professional skill, deployment plans should include scheduled periods without artificial intelligence, skills refreshers, and routine auditing of unaided clinician performance to detect and correct deskilling.

Equity must be designed in, evidenced, and monitored continuously. Every dataset and model should be accompanied by a transparent data statement describing who is represented, who is missing, how labels were created, and where bias might arise. Studies should collect and report demographics and social risk factors; examine subgroup performance by sex, age, ethnicity, socioeconomic status, disability, language, and digital access; and set parity bounds with mitigation plans when gaps appear. For home-based tools, low-bandwidth operation, multilingual and accessible interfaces, and offline modes should be treated as core features rather than afterthoughts. Device-loan or subsidy schemes, caregiver-training resources, and digital-literacy support are practical enablers that convert access into adherence and benefit.

Governance and engineering discipline determine whether promising prototypes become safe services. Protocols and reports should follow up-to-date artificial-intelligence-specific reporting guidance for prediction models and clinical trials; adaptive systems should declare change-control plans that specify what can change, how it will be validated, and when re-approval or re-evaluation is triggered; and cyber security should be treated as a clinical safety requirement, with a software bill of materials, vulnerability disclosure, and patching processes in place. Interoperability should be built from the start using Health Level Seven Fast Healthcare Interoperability Resources and Substitutable Medical Applications and Reusable Technologies on Fast Healthcare Interoperability Resources so that rehabilitation artificial intelligence integrates with electronic health records, scheduling, and therapy platforms; without this, systems remain pilot-bound and cannot support team-based care.

Health-system implementation should proceed as capacity extension, not as a shortcut. In the near term, the safest, highest-value uses are those that automate routine measurement, increase the volume and quality of task-specific practice, and allow clinicians to supervise more therapy at the same time. Service adoption should be gated by minimum criteria: assessor-blinded, dose-matched trials that achieve minimal-clinically-important-difference-level benefit; externally validated performance with a real-world performance gap within a prespecified margin across multiple sites; demonstrated subgroup fairness with credible mitigations; cost-effectiveness and budget impact that justify displacement of existing care; interoperability and cyber security readiness; and clear post-market surveillance plans. Where evidence is strongest, such as activity-level improvements in post-stroke upper-limb programmes wider deployment is justified under these guardrails. Where evidence is preliminary, such as aphasia therapy, paediatrics, or unsupervised musculoskeletal programmes investment should prioritise robust trials rather than replacement of standard care.

A practical research agenda follows from these principles. Priorities include adequately powered, assessor-blinded, dose-matched randomised trials with participation and quality-of-life endpoints; pragmatic, multi-site evaluations that measure the real-world performance gap and downstream care use; cost-utility and budget-impact analyses that incorporate the full lifecycle of devices, remote support, and cyber security; registries and living evidence maps that track what works, for whom, and in which settings; and transparent model documentation and data-access pathways that enable replication. Co-design with patients, caregivers, and clinicians especially people with disabilities and communities historically excluded from research should be embedded from problem selection through deployment and maintenance, alongside workforce training in artificial-intelligence literacy and safe supervision.

In summary, artificial intelligence can amplify rehabilitation when it measurably improves patient-important outcomes, translates reliably from laboratory to clinic and home, and reaches those most often left behind. The field should adopt an adjunct-first posture, using artificial intelligence to increase practice intensity, automate measurement, and extend therapist reach, while resisting premature substitution for proven care. Systems that meet translational, safety, equity, and economic thresholds should move into routine use with ongoing monitoring; systems that do not should remain in evaluation. This stance will allow innovation to accelerate access and independence without sacrificing rigour, safety, or fairness.

#### Strengths

This review uses a prespecified, transparent approach tailored to a complex, multi-modality field. The design as an umbrella review of reviews allows decision-makers to see the full landscape clinical effectiveness, real-world performance, safety, usability, equity, economics, and deployment rather than a narrow technology slice. The eligibility framework is anchored to a Population–Exposure–Outcome structure, which suits exposure-based questions and avoids forcing incomparable interventions into a single Population–Intervention–Comparator–Outcome frame. Searches span biomedical, allied health, and engineering databases, capturing both clinical and technical literature. Screening and full-text assessment followed explicit rules, with deduplication and blinded decision-tracking to reduce selection error. Overlap across reviews was handled transparently by mapping shared primary studies and describing redundancy, reducing double counting in interpretation. Data charting was built around constructs that matter to rehabilitation services alignment to the International Classification of Functioning, assessor blinding, dose symmetry and telemetry parity, minimum clinically important differences and responder definitions, external validation and calibration, and systematic capture of usability, adherence, and adverse events so that synthesis speaks directly to practice. Equity was integrated using a structured framework for subgroup description and access barriers, not treated as an afterthought. Model- and trial-reporting standards guided extraction, which improves comparability across heterogeneous sources. Finally, the findings are translated into an auditable, deployment-oriented framework, making the review useful to clinicians, commissioners, and regulators.

#### Limitations

Conclusions rest on secondary evidence. Many included reviews synthesize small, heterogeneous primary studies, and reporting gaps in those studies constrain what any overview can claim. Because the aim was mapping rather than effect adjudication, no formal methodological quality or risk-of-bias appraisal was undertaken; this avoids false precision across dissimilar designs, but it also means we do not grade certainty of effect. Clinical and methodological heterogeneity, together with asymmetric exposure measurement between trial arms, precluded quantitative re-meta-analysis; as a result, we summarise directions of effect rather than pooled magnitudes. Despite explicit overlap mapping, residual double counting of influential trials across reviews is possible. The search was limited to English-language publications and to sources indexed up to a fixed cut-off date; language and time-lag bias cannot be excluded. Definitions of “artificial intelligence” vary across reviews, and some “technology-assisted” interventions include robotics or virtual reality without a demonstrated learning component; this taxonomy drift may over- or under-attribute effects to artificial intelligence. Participant totals, minimum clinically important differences, responder proportions, adverse-event taxonomies, usability instruments, equity variables, external validation, and cost data were inconsistently reported across the underlying reviews, limiting the granularity of cross-review comparisons. We did not contact corresponding authors or extract primary-study data anew, so errors or omissions in the reviewed sources could propagate. The practice framework proposed here is derived from synthesis and requires prospective uptake testing and periodic revision as new evidence and regulations emerge.

## Conclusion

Artificial intelligence can expand rehabilitation, but the promise currently exceeds proof. Across reviews, the most reproducible signal is activity gains in post-stroke upper-limb programmes using technology-assisted; superiority for impairment and independence is inconsistent once exposure is matched and assessors are blinded. Translation to routine care is limited by a real-world performance gap, sparse external validation and calibration, and under-reporting of safety, usability, equity, and cost. We propose adoption framework that require minimal clinically important difference–anchored benefit, dose symmetry, blinded assessment, external validation with declared performance loss from laboratory to clinic and home, equity-by-design, economic value, interoperability, and regulatory and cybersecurity readiness. Future priorities are multi-site, pragmatic, assessor-blinded, dose-matched trials, living evidence and model documentation, and post-market monitoring. Meeting these conditions will allow artificial intelligence to extend access without sacrificing rigour or fairness.

## Data Availability

The original contributions presented in the study are included in the article/Supplementary Material, further inquiries can be directed to the corresponding author.

## Glossary

ADL: Activities of Daily Living
AE: Adverse Event
API: Application Programming Interface
ARAT: Action Research Arm Test
ASPECTS: Alberta Stroke Program Early CT Score
AUC: Area Under the ROC Curve
BCI: Brain–Computer Interface
BI: Barthel Index
CCA: Corrected Covered Area (review overlap metric)
CHEERS-2022: Consolidated Health Economic Evaluation Reporting Standards (2022)
CHEERS-AI: CHEERS extension for AI-specific economic reporting
CI: Confidence Interval
CNN: Convolutional Neural Network
CONSORT-AI: Consolidated Standards of Reporting Trials — AI extension
CRF: Case Report Form
CT: Computed Tomography
CTA: CT Angiography
CTP: CT Perfusion
CVDP: Coordinated Vulnerability Disclosure Program
DECIDE-AI: Reporting guideline for early clinical evaluation of AI decision support
DL: Deep Learning
EEG: Electroencephalography
EHR: Electronic Health Record
EQ-5D: EuroQol 5-Dimension questionnaire
EU AI Act: European Union Artificial Intelligence Act
FDA: US Food & Drug Administration
FES: Functional Electrical Stimulation
FHIR (HL7 FHIR): Fast Healthcare Interoperability Resources
FMA-UE: Fugl–Meyer Assessment of the Upper Extremity
HL7: Health Level Seven (standards body)
ICER: Incremental Cost-Effectiveness Ratio
ICF: International Classification of Functioning, Disability and Health
IMU: Inertial Measurement Unit
ITR: Information Transfer Rate (BCI)
JBI: Joanna Briggs Institute
LLM: Large Language Model
MAE: Mean Absolute Error
MAD: Median Absolute Deviation
MBI: Modified Barthel Index
MCID: Minimal Clinically Important Difference
ML: Machine Learning
MMSE: Mini-Mental State Examination
mRS: Modified Rankin Scale
NLP: Natural Language Processing
PCCP (FDA): Predetermined Change Control Plan
PEO: Population–Exposure–Outcome
PICO: Population–Intervention–Comparator–Outcome
PRIOR: Preferred Reporting Items for Overviews of Reviews
PRISMA-ScR: PRISMA extension for Scoping Reviews
PROBAST-AI: Prediction model Risk Of Bias ASsessment Tool — AI extension
PROGRESS-Plus: Equity framework: Place of residence, Race/ethnicity/culture/language, Occupation, Gender/sex, Religion, Education, Socioeconomic status, Social capital + context factors
QALY: Quality-Adjusted Life-Year
RCT: Randomised Controlled Trial
RMSE: Root Mean Square Error
SAP: Statistical Analysis Plan
SBOM: Software Bill of Materials
SMART-on-FHIR: Substitutable Medical Apps, Reusable Technologies on FHIR
SLA: Service-Level Agreement
SOP: Standard Operating Procedure
SPIRIT-AI: Standard Protocol Items — Recommendations for Interventional Trials — AI extension
SUCRA: Surface Under the Cumulative Ranking curve (network meta-analysis)
SWiM: Synthesis Without Meta-analysis (reporting guideline)
TEFCA: Trusted Exchange Framework and Common Agreement (U.S.)
UEQ: User Experience Questionnaire
V&V: Verification and Validation
VEP: Visual-Evoked Potential
VR/AR: Virtual Reality/Augmented Reality
WMFT: Wolf Motor Function Test
WHO, World Health Organization: ΔReal, Lab-to-clinic-to-home generalisation delta (pre-specified real-world performance loss).
