# Supplementary material for: Artificial intelligence in rehabilitation: a review of clinical effectiveness, real-world performance, safety, and equity across modalities and settings
Source: Front Digit Health. 2026 Mar 18;8:1737957. doi: 10.3389/fdgth.2026.1737957 (PMC13040452; doi:10.3389/fdgth.2026.1737957)
Supplement: Supplementary file 1 [file Table1.docx]

Supplementary Table 1: Search Strategy

| Database | Query |
| --- | --- |
| MEDLINE via Ovid | 1. exp Rehabilitation/ OR exp Physical Therapy Modalities/ OR exp Occupational Therapy/ OR exp Speech Therapy/ OR exp Stroke Rehabilitation/ OR Telerehabilitation/ 2. exp Artificial Intelligence/ OR exp Machine Learning/ OR Deep Learning/ OR Robotics/ OR Virtual Reality/ OR Natural Language Processing/ OR Brain-Computer Interfaces/ OR Computer Vision/ 3. ("artificial intelligence" OR "machine learning" OR "deep learning" OR algorithm* OR "neural network*" OR "computer vision" OR "pose estimation" OR skeleton* OR exoskeleton* OR robot* OR "virtual reality" OR "augmented reality" OR "mixed reality" OR "extended reality" OR "brain computer interface*" OR BCI OR "functional electrical stimulation" OR FES OR "natural language processing" OR NLP OR "large language model*" OR LLM* OR chatbot* OR "conversational agent*" OR wearable* OR sensor* OR IMU OR electromyograph* OR EMG OR sEMG OR acceleromet* OR gyroscop* OR radiomics OR "decision support" OR prognos* OR predict*).ti,ab,kf. 4. (rehabilitat* OR "physical therap*" OR physiotherap* OR "occupational therap*" OR "speech therap*" OR "speech and language therap*" OR neurorehabilitat* OR telerehabilitat* OR gait OR ambulation OR "upper limb" OR "upper extremity" OR "lower limb" OR "lower extremity" OR dexterity OR "hand function" OR "activities of daily living" OR ADL OR participation OR "cognitive rehab*" OR aphasia OR "balance training" OR "postural control" OR "stroke rehab*").ti,ab,kf. 5. 2 OR 3 6. 1 OR 4 7. 5 AND 6 8. (meta analysis.pt. OR systematic review.pt. OR (meta-analy* OR "systematic review*" OR "scoping review*" OR "umbrella review*" OR "overview of reviews" OR "evidence map*" OR review*).ti,ab.) 9. 7 AND 8 10. limit 9 to (english language AND humans) 11. limit 10 to yr="1946 - 2025" |
| Embase via Ovid | 1. exp rehabilitation/ OR exp physiotherapy/ OR exp occupational therapy/ OR exp speech therapy/ OR stroke rehabilitation/ OR telerehabilitation/ 2. exp artificial intelligence/ OR exp machine learning/ OR deep learning/ OR robot/ OR virtual reality/ OR natural language processing/ OR brain computer interface/ OR computer vision/ 3. ("artificial intelligence" OR "machine learning" OR "deep learning" OR algorithm* OR "neural network*" OR "computer vision" OR "pose estimation" OR skeleton* OR exoskeleton* OR robot* OR "virtual reality" OR "augmented reality" OR "mixed reality" OR "extended reality" OR "brain computer interface*" OR BCI OR "functional electrical stimulation" OR FES OR "natural language processing" OR NLP OR "large language model*" OR LLM* OR chatbot* OR "conversational agent*" OR wearable* OR sensor* OR IMU OR electromyograph* OR EMG OR sEMG OR acceleromet* OR gyroscop* OR radiomics OR "decision support" OR prognos* OR predict*).ti,ab,kw. 4. (rehabilitat* OR "physical therap*" OR physiotherap* OR "occupational therap*" OR "speech therap*" OR "speech and language therap*" OR neurorehabilitat* OR telerehabilitat* OR gait OR ambulation OR "upper limb" OR "upper extremity" OR "lower limb" OR "lower extremity" OR dexterity OR "hand function" OR "activities of daily living" OR ADL OR participation OR "cognitive rehab*" OR aphasia OR "balance training" OR "postural control" OR "stroke rehab*").ti,ab,kw. 5. 2 OR 3 6. 1 OR 4 7. 5 AND 6 8. (systematic review/ OR meta analysis/ OR review/ OR (meta-analy* OR "systematic review*" OR "scoping review*" OR "umbrella review*" OR "overview of reviews" OR "evidence map*" OR review*).ti,ab.) 9. 7 AND 8 10. limit 9 to (human AND english language) 11. limit 10 to yr="1947 - 2025" |
| Web of Science Core Collection | TS=(rehabilitat* OR "physical therap*" OR physiotherap* OR "occupational therap*" OR "speech therap*" OR "speech and language therap*" OR neurorehabilitat* OR telerehabilitat* OR gait OR ambulation OR "upper limb" OR "upper extremity" OR "lower limb" OR "lower extremity" OR dexterity OR "hand function" OR "activities of daily living" OR ADL OR participation OR "cognitive rehab*" OR aphasia OR "balance training" OR "postural control" OR "stroke rehab*") NEAR/3 TS=("artificial intelligence" OR "machine learning" OR "deep learning" OR algorithm* OR "neural network*" OR "computer vision" OR "pose estimation" OR skeleton* OR exoskeleton* OR robot* OR "virtual reality" OR "augmented reality" OR "mixed reality" OR "extended reality" OR "brain computer interface*" OR BCI OR "functional electrical stimulation" OR FES OR "natural language processing" OR NLP OR "large language model*" OR LLM* OR chatbot* OR "conversational agent*" OR wearable* OR sensor* OR IMU OR electromyograph* OR EMG OR sEMG OR acceleromet* OR gyroscop* OR radiomics OR "decision support" OR prognos* OR predict*) AND LA=(English) AND DT=(Review) AND PY=(1900-2025) |
| Scopus | ( TITLE-ABS-KEY(rehabilitat* OR "physical therap*" OR physiotherap* OR "occupational therap*" OR "speech therap*" OR "speech and language therap*" OR neurorehabilitat* OR telerehabilitat* OR gait OR ambulation OR "upper limb" OR "upper extremity" OR "lower limb" OR "lower extremity" OR dexterity OR "hand function" OR "activities of daily living" OR ADL OR participation OR "cognitive rehab*" OR aphasia OR "balance training" OR "postural control" OR "stroke rehab*") W/3 TITLE-ABS-KEY("artificial intelligence" OR "machine learning" OR "deep learning" OR algorithm* OR "neural network*" OR "computer vision" OR "pose estimation" OR skeleton* OR exoskeleton* OR robot* OR "virtual reality" OR "augmented reality" OR "mixed reality" OR "extended reality" OR "brain computer interface*" OR BCI OR "functional electrical stimulation" OR FES OR "natural language processing" OR NLP OR "large language model*" OR LLM* OR chatbot* OR "conversational agent*" OR wearable* OR sensor* OR IMU OR electromyograph* OR EMG OR sEMG OR acceleromet* OR gyroscop* OR radiomics OR "decision support" OR prognos* OR predict*) ) AND ( DOCTYPE(re) OR DOCTYPE(cr) OR TITLE-ABS-KEY(meta-analy* OR "systematic review*" OR "scoping review*" OR "umbrella review*" OR "overview of reviews" OR "evidence map*" OR review*) ) AND ( LIMIT-TO ( LANGUAGE, "English" ) ) AND ( PUBYEAR <= 2025 ) |
| CINAHL via EBSCO | S1 MH "Rehabilitation+" OR MH "Physical Therapy+" OR MH "Occupational Therapy+" OR MH "Speech Therapy+" OR MH "Stroke Rehabilitation" OR MH "Telerehabilitation" S2 MH "Artificial Intelligence" OR MH "Machine Learning" OR MH "Robotics" OR MH "Virtual Reality" OR MH "Brain-Computer Interfaces" S3 TI (rehabilitat* OR "physical therap*" OR physiotherap* OR "occupational therap*" OR "speech therap*" OR "speech and language therap*" OR neurorehabilitat* OR telerehabilitat* OR gait OR ambulation OR "upper limb" OR "upper extremity" OR "lower limb" OR "lower extremity" OR dexterity OR "hand function" OR "activities of daily living" OR ADL OR participation OR "cognitive rehab*" OR aphasia OR "balance training" OR "postural control" OR "stroke rehab*") OR AB (same terms) S4 TI ("artificial intelligence" OR "machine learning" OR "deep learning" OR algorithm* OR "neural network*" OR "computer vision" OR "pose estimation" OR skeleton* OR exoskeleton* OR robot* OR "virtual reality" OR "augmented reality" OR "mixed reality" OR "extended reality" OR "brain computer interface*" OR BCI OR "functional electrical stimulation" OR FES OR "natural language processing" OR NLP OR "large language model*" OR LLM* OR chatbot* OR "conversational agent*" OR wearable* OR sensor* OR IMU OR electromyograph* OR EMG OR sEMG OR acceleromet* OR gyroscop* OR radiomics OR "decision support" OR prognos* OR predict*) OR AB (same terms) S5 S2 OR S4 S6 S1 OR S3 S7 S5 AND S6 S8 PT "Systematic Review" OR PT "Meta Analysis" OR PT "Review" OR TI (meta-analy* OR "systematic review*" OR "scoping review*" OR "umbrella review*" OR "overview of reviews" OR "evidence map*" OR review*) OR AB (same terms) S9 S7 AND S8 Limiters: English; Human; Publication Date through 2025-09-01 |
| IEEE Xplore | ( Abstract:(rehabilitat* OR "physical therapy" OR physiotherapy OR "occupational therapy" OR neurorehabilitation OR telerehabilitation OR gait OR ambulation OR "upper limb" OR "lower limb" OR "hand function" OR "activities of daily living" OR ADL OR participation OR "cognitive rehab" OR aphasia OR "balance training" OR "postural control" OR "stroke rehab") ) AND ( Abstract:("artificial intelligence" OR "machine learning" OR "deep learning" OR algorithm* OR "neural network*" OR "computer vision" OR "pose estimation" OR skeleton* OR exoskeleton* OR robotics OR "virtual reality" OR "augmented reality" OR "mixed reality" OR "extended reality" OR "brain computer interface" OR chatbot OR "large language model" OR wearable OR sensor OR IMU OR EMG OR sEMG OR accelerometer OR gyroscope OR radiomics OR "decision support" OR prognosis OR prediction) ) AND ( Abstract:(review OR survey) ) Content Type: Journals; Conferences Language: English Date range: Inception–2025-09-01 |
