# Supplementary material for: Artificial intelligence in rehabilitation: a review of clinical effectiveness, real-world performance, safety, and equity across modalities and settings
Source: Front Digit Health. 2026 Mar 18;8:1737957. doi: 10.3389/fdgth.2026.1737957 (PMC13040452; doi:10.3389/fdgth.2026.1737957)
Supplement: Supplementary file 2 [file Table2.docx]

Supplementary Table 2

| Authors (et al.) | Review type | Primary AI modalities | Rehabilitation context | N (studies / participants) | Key outcomes | AI classification |
| --- | --- | --- | --- | --- | --- | --- |
| Sardari et al., 2023 | Systematic review (PRISMA-guided) of AI for skeleton-based action evaluation in physical rehabilitation | Vision-based skeleton/joint analysis; DL pipelines (CNN, LSTM, GCN, ST-GCN); feature learning and automated scoring/feedback | \|  \| \| --- \|  \| Mixed settings (clinic/home); tasks span general rehab and disease-specific exercises; datasets and evaluations are largely lab-centric with limited real-world/home generalization. \| \| --- \| | 32 / dataset-specific (not pooled)—review collates algorithmic studies and public datasets rather than aggregating patient totals | No clinical effect meta-analysis; synthesizes model performance: e.g., MAD 0.025 (UI-PRMD) and 0.038 (KIMORE), RMSE LSTM-GCN 0.191 vs LSTM-HF 0.290, MAE 0.021 vs 0.025 (UI-PRMD), and ~98% gesture-level classification in patient identification (IRDS). Highlights gaps in explainability/feedback and dataset limitations (size, realism, sensor constraints). Performance figures often come from non-subject-wise or single-view splits; errors increase under subject-wise/cross-view validation. | AI‑enabled (ML-based) |
| Yang R., et al. (2024) | Scientometric/bibliometric review (CiteSpace, WOS 2002–2022) | Field-level AI landscape across CV/ML/DL, NLP, intelligent robotics, tele-rehab; maps research clusters/hotspots | Rehabilitation science broadly (injury diagnosis, sports medicine, neuro-rehab, tele-rehab); not a clinical-effectiveness review | 1,499 publications; participants N/A (bibliometric) | Exponential growth since 2017 (peak 2021); US leads output; identifies nine hot topics and influential works; emphasizes emerging focus on deep learning/computer vision in rehab science | AI‑enabled (ML-based) |
| Sumner et al., 2023 | Systematic review of AI-supported physical rehabilitation evaluated in clinical settings | App-based systems; robotics (restore/replace function); gaming/VR; wearables | Adults in formal rehabilitation; settings: home (n=14), clinic (n=10), mixed (n=4); conditions mainly stroke, back/neck pain, PD, limb absence | 28 projects (29 publications) / participants ranged 1–461 (5 RCTs) | Outcomes grouped as physical function/activity, pain, HRQoL; clinical effects inconsistent; ROB2: 3 studies “some concerns,” 2 high risk of bias; implementation enablers (access, remote monitoring, lower manpower) and barriers (tech literacy, reliability, fatigue). None of the included interventions reported regulatory certification for clinical use. | Technology‑assisted (no ML demonstrated) |
| **Reis FJJ, et al., 2024** | **Scoping review** (JBI methodology; **PRISMA-ScR**; OSF-registered protocol) | Clinical **machine learning** across supervised/unsupervised approaches; **44** model families identified—most frequently **Random Forest**, **SVM**, **CNN**; robotics/VR **excluded** by design. | Clinical **physical therapy** (primary–tertiary care) spanning **musculoskeletal, neurological, sports**; ML applied to **diagnosis, prognosis, treatment-outcome prediction, clinical decision support, movement analysis, patient monitoring, personalized care**. | **42 / N/A** (participants not aggregated in scoping synthesis). | Field mapping shows rapid post-2020 growth; **limited model availability** (publicly available models in **3/42**; overall availability reporting sparse); frequent under-reporting of **performance metrics, costs, interpretability/explainability**; calls for validated, transparent models prior to clinical deployment. | AI-enabled (ML-based) |
| Antoni T., et al. (2025) | Systematic review & meta-analysis of RCTs (2009–2024) comparing AI-assisted vs conventional post-stroke rehab; PRISMA-guided. | Robotic systems (end-effector, exoskeleton) and robot+VR combinations; adaptive, AI-assisted feedback across platforms. | Post-stroke rehab—predominantly subacute (3–6 months); one trial included chronic (≥12 months) patients. | 7 RCTs / 213 participants (6 included in meta-analysis for BI/MI). | No superiority vs conventional care on: Barthel Index SMD 0.16 (95% CI −0.08 to 0.39; I² = 0%) and Motricity Index SMD 0.60 (−0.10 to 1.30; I² = 86%); Authors claim non-inferiority using external margins, but this approach does not establish non-inferiority (no prespecified margin/CI testing on between-group differences). Overall RoB mostly low with some concerns in randomization/blinding. | Technology‑assisted (no ML demonstrated) |
| Zhichao & Yu, 2025 | Narrative review mapping the past–present–future of AI in stroke rehabilitation | Robotics, VR/AR, BCI, wearable sensors & IoT, ML/DL, multimodal imaging (CT/MRI/PET; OCT), cloud/tele-rehab | Stroke motor, cognitive, language, psychological & social rehab; hospital + home/remote; spans acute → chronic | Narrative overview; no pooled N | Synthesizes evidence and trends: reports meta-analytic signals of benefit for robot-assisted rehab; emphasizes personalization/precision, remote & home programs, and multimodal integration; flags data-standardization, generalizability, and privacy/ethics/black-box risks; recommends interdisciplinary development, portable sensors, and explainable AI for clinical deployment. | Mixed (AI + technology‑assisted) |
| Rahman S., et al. (2023) | Systematic review (PRISMA-adapted), covering automated post-stroke rehab systems and assessment (2013→). | Robot-assisted and VR-based systems; data-driven assessment with deep learning on spatio-temporal skeleton data (incl. dynamic attention). | Post-stroke rehabilitation across inpatient and outpatient/home settings | 48 publications (24 robot/VR systems; 17 ML assessment; 7 datasets) / N/A (participants not pooled). | Benchmarked methods on KIMORE and UI-PRMD; automated methods show high agreement with therapists; DL with skeleton + dynamic attention outperforms others (e.g., best avg MAD 0.576 on KIMORE); reported RMSE as low as 0.55. Highlights gaps: limited public datasets, non-uniform assessment, cost/safety issues. | Mixed (AI + technology‑assisted) |
| Kapil D., et al. (2025) | Systematic review & meta-analysis of RCTs (PRISMA), AI-assisted physiotherapy vs usual care for non-specific low back pain (NSLBP) | Predominantly AI-guided mHealth apps (e.g., tailored self-management, interactive education/feedback), wearable-linked monitoring/feedback, and an AI-calibrated resistance trainer | Adults with NSLBP across primary/secondary care and home-based programs; intervention duration 4 weeks to 6 months | 8 studies / 2,147 participants (6 trials meta-analysed) | No significant added benefit vs usual physiotherapy on pooled outcomes: pain, functional impairment, mental health; moderate heterogeneity for function; risk-of-bias mainly from unavoidable blinding limitations; no clear publication bias on Egger tests. One pooled result shows a CI apparently excluding zero with a non-significant p-value; treat as a reporting/analysis inconsistency and interpret cautiously. | Technology‑assisted (no ML demonstrated) |
| **Boltaboyeva A., et al. (2025)** | **Narrative review** (targeted search across 2018–2025; multi-database) | **ML/DL** for movement/physiology analysis; **IoT/wearable & ambient sensors** (ECG/PPG/IMU/UWB, sEMG, BCG); **LLM-powered virtual assistants/chatbots**; integrated **telerehab/VR** platforms | Broad **medical rehabilitation** across neuro/musculoskeletal and post-surgical contexts; **home/remote + inpatient** monitoring; emphasis on **sensor-based recovery tracking** and platform integration | **112 / N/A** | Proposes **integrated AI–IoT–LLM architecture**; maps leading platforms/sensors and ML performance; highlights gains in remote monitoring and personalization; flags **heterogeneity, privacy, interoperability, and limited clinical validation**; calls for **standards, external validation, and scalable, privacy-preserving deployment**. | Mixed (AI + technology‑assisted) |
| Lanotte F., et al. (2023) | Narrative review / perspective proposing an end-to-end framework for AI in rehabilitation | EHR-driven ML (screening/prognosis); medical-imaging DL; wearable sensors (IMU/EMG/ECG); instrumented environments / ambient intelligence; video-based pose estimation; early use of LLMs for clinical text | Cross-cutting across screening → diagnosis → treatment → monitoring; IRF, outpatient, home, community; neuro & musculoskeletal; acute → chronic phases | Narrative (no pooled N) | Identifies three barriers (interoperability, transparency, actionability) and a 7-step development framework (data configuration → external validation); showcases examples where sensors + AI improve post-stroke walking prediction and video/pose enables automated scoring; emphasizes standardization, fairness/ subgroup checks, clinician-in-the-loop deployment, and external validation before scale-up. | Mixed (AI + technology‑assisted) |
| Calderone A., et al. (2024) | Systematic review (PRISMA; OSF-registered) | ML/DL, computer vision, robotics/exoskeletons, VR, BCI, wearables/smart devices, XAI | Neurorehabilitation & diagnosis across stroke, SCI, PD; inpatient, outpatient/home; acute → chronic | 8 / N/A (participants not pooled) | Signals of benefit and feasibility across modalities: RCT showed **EMG-AI robotic hand** improved **FMA** and spasticity in chronic stroke; CV/ML models aligned with clinician **PD gait** ratings and enabled **remote** assessment; **nocturnal-breathing AI** detected/monitored PD with high accuracy; **ML/XAI** outperformed classical stats for **post-stroke upper-limb recovery** prediction; **SCI gait** prediction accurate (FAC at discharge); **stroke proprioception** classification via ML surpassed task scores. Heterogeneity and limited long-term/clinical validation noted; calls for larger trials, explainability, privacy safeguards, and home-based access. | Mixed (AI + technology‑assisted) |
| Zhang Y., et al. (2021) | \|  \| \| --- \|  \| Narrative review / perspective on AI for myasthenia gravis (MG) rehabilitation & long-term management \| \| --- \| | ML/DL decision support; EHR-integrated digital platforms; remote monitoring via wearables & video; telerehab/app-based adherence; AI trial-matching | MG chronic self-management in outpatient/home settings (medication safety, lifestyle/exercise, work/school return); proposes AI-enabled AE monitoring, education, and clinician-patient social tools | Narrative—no pooled N | Concept roadmap rather than effect synthesis: calls for AI-driven AE monitoring, patient education, doctor–patient social platform, and AI-assisted recruitment; advocates continuous data capture with sensors/apps/video to overcome MG trial barriers (recruitment, supervision, outcomes); notes low-quality exercise evidence in MG and need for standardized outcomes and remote-supervised protocols. | Mixed (AI + technology‑assisted) |
| **Mulpuri R.P., et al. (2024)** | **Systematic review (PRISMA 2020)** of AI/ML in **neuroregeneration** | **Deep learning (41%)**, classic **ML (29%)**, **radiomics (12%)**, **robotic/targeted stimulation (12%)**, BCI/neuroprosthetics; imaging- and EHR-based models | **Neuroregeneration across PNS/CNS** (peripheral nerve injury, SCI, neurodegenerative conditions); includes **robotic neurorehab** and **stimulation** for functional recovery; mostly **preclinical/early clinical** | **19 / N/A** (mixed designs; participants not pooled) | Dominant aims: **diagnosis/prognosis** and **robotic rehab**; many models reported **high performance** (e.g., accuracy ≳90%, AUC ≳0.90). Reports **15–20% functional gains** and ~**20% nerve-conduction** improvements in robotic/stimulation studies; **AI-driven drug discovery** showed **50–100% neurite-length** gains in preclinical work. **Methodological heterogeneity** and reporting gaps noted; calls for rigorous design/validation. | Mixed (AI + technology‑assisted) |
| Attoh-Mensah E., et al. (2025) | Mini narrative review + SWOT on AI for personalized rehabilitation (current applications and barriers/enablers) | LLM/chatbots (ChatGPT 3.5/4) for plan generation & support; ML/DL on data streams; wearables/IMUs, motion-tracking & physio monitors for just-in-time adaptive dosing; telerehab platforms | Broad: stroke, musculoskeletal, chronic pain, cardiac, neurocognitive; clinic + home/remote; pipeline spans plan generation → ongoing management → real-time adaptation | Narrative (no pooled N)—evidence base largely protocols, case/observational, early trials | SWOT synthesis: *Strengths*—personalization at scale, automation to reduce error/workload; *Weaknesses*—adoption resistance, costs, interoperability, accuracy/quality gaps; *Opportunities*—aging-driven demand, telerehab reach, on-device/open-source AI, cross-institution data sharing; *Threats*—privacy/security risk, bias and inequity amplification, over-reliance, regulatory constraints. Overall: promising but early-stage; calls for rigorous validation, explainability, and balanced regulation. | Mixed (AI + technology‑assisted) |
| **Zhu Y., et al. (2023)** | **Network meta-analysis of RCTs** (systematic review; PRISMA 2020; PROSPERO **CRD42022337776**) | **RT, IR, RT+VR, VR, BCI, RR** (six AI rehab modalities) | **Post-stroke** upper-limb dysfunction; outcomes: **FMA-UE Total/Proximal/Distal, ARAT, MBI** | **101** RCTs / **4,702** participants. | SUCRA rankings: RT+VR highest for FMA-UE Proximal/Distal and ARAT; IR highest for FMA-UE Total; BCI highest for MBI. Note: SUCRA conveys probabilistic ranking under model assumptions and does not imply clinical superiority; pairwise contrasts for FMA-UE Total/Distal and MBI were generally non-significant. | Technology‑assisted (no ML demonstrated) |
| **Zhao Z., et al. (2024)** | **Narrative review** of **AI for neuroimaging** in ischemic stroke rehab/prognosis (CT/CTA/CTP & MRI focus). | **ML/DL** (CNN/ResNet; radiomics), **e-ASPECTS**, automated **perfusion** software; combined imaging+clinical models. | **Ischemic stroke**—prediction of outcomes (mRS, infarct volume) to guide **acute → post-acute rehab planning** and patient selection (e.g., thrombectomy). | **49 / N/A** (bibliographic synthesis; participants not pooled). | DL often **outperforms** traditional methods: functional-outcome AUC ~**0.71**; reperfusion AUC ~**0.65**; **radiomics+ASPECTS** for malignant MCA infarction AUC **0.917–0.913**; **CTA-venous ASPECTS** correlates with 90-day mRS (AUC **0.82**); DL on CTP predicts final infarct **better than RAPID** (lower MAD); accuracy drops for **very large infarcts**; overall promise for **prognosis & rehab planning** but needs external validation and standardization. | AI‑enabled (ML-based) |
| **Gutierrez-Martinez J., et al. (2021)** | **Systematic review (PRISMA)** of **visual-evoked-potential BCIs** (P300/SSVEP) for motor rehabilitation, emphasizing AI classifiers and performance reporting. | Dominant **ML**: **LDA (~49%)**, **SVM (~16%)**; **DL** rare (single **CNN** study); also **FBCCA/BLDA** variants; hybrid (P300+SSVEP/MI) explored. | Motor-rehab control of FES, orthosis/exoskeleton, prosthesis, and VR; mainly lab studies, only 4 with patients (e.g., SCI/ALS). Hand and lower-limb tasks predominate. Only 4 of 34 included studies enrolled patient cohorts (most were healthy-volunteer, offline evaluations). | **34 / N/A** (participants not pooled). | Reported **accuracy 38–100%**; **ITR 1.55–49.25 bits/min**; modality split **SSVEP 55.8%**, **P300 26.47%**, **hybrid 17.64%**; application mix: **Orthosis 29.41%**, **VR 23.52%**, **FES 17.64%**, **RRS 17.64%**, **Prosthesis 11.76%**. Under-reporting of metrics/validation common; future work: optimize visual stimuli and training for **online rehab BCIs**. | AI‑enabled (ML-based) |
| **Adikari A., et al. (2024)** | **Scoping review** (PRISMA-ScR) mapping AI in aphasia across assessment, therapy, and self-management | **Supervised ML** (incl. DL: CNN/RNN), **unsupervised ML**, **NLP** (language models/chatbots), **fuzzy rules**, **genetic programming**; some **VR-based** therapy tools | **Aphasia** (mostly post-stroke) in **speech-language pathology**—diagnosis/classification, therapy feedback, assistive/self-management; clinic & tele/at-home contexts | **77 / N/A** (participants not pooled) | Field has moved from early prototypes → automated **assessment** dominance to emerging **therapy** and **personalised assistive systems**; **69%** supervised ML; **NLP** use rising; limited unsupervised learning; major gaps in annotated datasets, clinical deployment, and long-term validation; opportunities in multimodal data fusion and LLM-enabled therapy/chatbots. | Mixed (AI + technology‑assisted) |
| **Khalid U., et al. (2024)** | Structured **narrative review** with PRISMA-guided search/selection (2020–2023) | **ML/DL** (incl. **reinforcement learning**), **VR/AR**, **social & assistive robots**, **wearables/IoT** sensors, **chatbots/NLP**, **telerehabilitation** platforms | Predominantly **cognitive rehab** across **neurocognitive/neurodegenerative** (MCI, dementia/AD, PD), **neurodevelopmental** (ASD, ADHD), and **post-stroke**; clinic + **home/tele**; also **cardio-oncology** and **diabetes** telerehab | **Survey (2020–2023) / N/A** (participants not pooled) | AI shows **accessibility & personalization** gains (e.g., **RL-adaptive** CRT, VR exergaming, sensor-guided home programs); **uptake growing** since 2020 with journals ≈**92%** of sources; persistent gaps in **validation**, **data quality/representativeness**, **privacy**, and **clinical training**; emphasis that AI should **augment—not replace—clinicians**; calls for standards, external validation, and equity checks before scale-up. | Mixed (AI + technology‑assisted) |
| Chandrabhatla A.S., et al. (2023) | Narrative review of FDA-approved AI/ML technologies for stroke (diagnosis + rehab) | Predominantly CNN-based radiology triage/quantification (CTA LVO, CTP core/penumbra, ASPECTS, ICH); plus neuromodulatory/BCI devices (BrainQ, IpsiHand) | post-stroke rehabilitation devices (IpsiHand—FDA De Novo clearance, 2021; BrainQ—FDA Breakthrough Device designation, not clearance) alongside acute imaging-workflow tools; rehab indicated for post-stroke neurological/functional recovery. | 22 FDA-approved technologies synthesized; results summarized from 45 primary publications (participants not pooled) | Diagnostic AI comparable to neuroradiologists; improves workflow and some patient outcomes. Examples: Viz LVO AUC 0.91 and reduced scan-to-notification from 58→7 min; reduced transfer/notification times and Neuro-ICU LOS (−2.5 days) . CTP tools: Vitrea often outperforms RAPID for final infarct volume prediction; strong correlations reported across platforms . ICH: BriefCase cut outpatient interpretation delays by 90% (−604 min) and inpatient by 38 min; NPV 96–99%, PPV 72–96% | Mixed (AI + technology‑assisted) |
| Kaelin V.C., et al. (2021) | Scoping review (PRISMA-ScR; OSF-registered protocol) examining AI within pediatric rehab interventions targeting participation. | Multiple AI types, most commonly robotics (72/94; 77%), HMI (51/94; 54%), plus VR (19/94; 20%), NLP (18/94; 19%), ML (11/94; 12%), computer vision (10/94; 11%). | Pediatric participation across diagnoses (esp. ASD 46%, CP 19%); delivery mainly in-person (84/94; 89%), with remote 11%. | 94 / N/A (participants not pooled); reported sample sizes 1–120, mean ≈14 per study. | Field map + gaps: overwhelming in-person/robotics focus; categorical (group) personalization 99% vs individuated 1%; goal-setting present in only 1%; calls for remote delivery, individual goal-setting, and tailoring to individually reported participation needs. | Mixed (AI + technology‑assisted) |
| **Nambi G., et al. (2024)** | **Practice-based narrative review** on clinical usefulness of AI in **physiotherapy** | **ML/DL prediction models** (e.g., hip-fracture prognosis), **computer vision/gait & posture analysis** (incl. thermography), **NLP/chatbots** (e.g., “physioGPT”), **decision-support** and workflow optimization; **wearables/remote monitoring** | Broad physiotherapy across **musculoskeletal & neurological** conditions; **assessment→planning→follow-up** in **outpatient/ward & home/tele** contexts | **Narrative—no pooled N** | Synthesizes benefits (more **precise assessment**, **personalized plans**, scheduling and workflow gains) and risks (**privacy**, **over-reliance**, **costs**). Notes **limited PT knowledge/uptake**, cost/availability as key barriers, and calls for **clear governance**, **robust evaluation metrics**, and use of AI to **augment, not replace, clinicians**. | AI‑enabled (ML-based) |
| **Balgude S.D., et al. (2024)** | **Systematic literature review (PRISMA flow)** on AI/ML for **cerebral palsy** diagnosis, prognosis & management (2014–2024) | **ML/DL** across classic classifiers (**SVM, LDA, DT, kNN, LR, ensembles**) and deep nets (**CNN, LSTM, GCN**); **video pose-estimation** pipelines; datasets (MINI-RGBD, RVI, MODYS-video) | **CP across the care pathway:** early prediction (GMA video), diagnosis/classification, treatment & rehab support; pediatric clinic + **remote/tele** assessment | **69 / N/A** (participants not pooled) | Maps methods, datasets, and metrics; reports promising model performance in early-risk screening and movement assessment; identifies **small datasets, heterogeneity, limited external validation, and implementation/ethics gaps**; calls for **standardized datasets, explainability, and clinical integration**. | AI‑enabled (ML-based) |
| **Kholiya K. (2024)** | **Narrative/Concept paper with proposed mixed-methods evaluation** (literature review + case studies + interviews/surveys) | **AI-driven personalization** of treatment plans, **real-time monitoring** via sensors/wearables, and **decision-support** for clinicians; computer-vision–assisted assessment discussed conceptually. | Broad **physiotherapy practice** (clinic/rehab services), positioned as a pathway to more **patient-centred**, data-driven rehab across conditions. | **N/A** (concept/methods paper; no enumerated included studies or pooled participants). | Claims of **improved PROs, functional metrics, and adherence**, alongside positive clinician experience; emphasizes ethics (privacy/bias) and calls for rigorous evaluation/validation. | Unclear / narrative (no ML demonstrated) |
| **Nicora G., et al. (2025)** | **Systematic review** (PRISMA-style) of AI/ML in **robotic-assisted rehabilitation** across motor **and** neurocognitive domains; human-participant studies from Scopus & IEEE Xplore. | **NN/DL** (CNN, LSTM/RNN), **SVM/k-NN/RF**, **reinforcement learning**, fuzzy NN; inputs from **robot sensors, EMG/EEG, IMU/kinematics**; online & offline control/assessment pipelines. | **Multi-domain robotic rehab** (upper/lower limb, gait, exoskeletons/end-effectors; inpatient, outpatient, home). Tasks include **movement/intention classification, trajectory prediction, patient assessment, compensation detection, motivation/engagement**. | **201 / N/A** (participants not pooled). | ML is **effective** for intent prediction, assessment (e.g., FMA/6MWD prediction), and compensation detection; however, **72%** of studies used **healthy** subjects; **offline→online** performance **drops**; **generalization to patients degrades**; **limited** data/code sharing and inconsistent reporting (few cross-subject designs). Calls for **XAI**, **benchmarks**, external validation, and performance **monitoring** in deployment. | Mixed (AI + technology‑assisted) |
| **Abedi A., et al. (2024)** | **Scoping review (PRISMA-ScR; Arksey & O’Malley)** of **AI-driven virtual/tele-rehabilitation at home** (adult patients; to June 2023). | Predominantly **fuzzy rule-based methods**, **template matching**, and **deep neural networks**; also ANFIS/ML variants; inputs from **RGB/Kinect**, **IMU/wearables**, and **exoskeleton sensors**. | **Home-based VRehab** across conditions (majority **stroke**; also orthopedic, cardiac, Rett syndrome); programs deliver **exercise assessment + real-time feedback** and remote monitoring. | **13 / 276** (139F/137M across included studies). | Evidence suggests **improved outcomes** vs in-person or non-AI VRehab in several studies (e.g., gains in **FMA**, **Wolf Motor Function Test**, **Mobility Index/Brunnstrom**, **lower readmissions**, **high satisfaction**), but the literature is **sparse and heterogeneous**; calls for **standardized metrics**, **validated usability/safety scales**, **co-design**, and **privacy-preserving personalization** (e.g., federated learning) before scale-up. | Mixed (AI + technology‑assisted) |
| **Vélez-Guerrero M.A., et al. (2021)** | **Systematic review (PRISMA)** of **AI-based wearable/mobile upper-limb exoskeletons** (2016–2020) | **ANNs (~40%)**, **adaptive algorithms (~20%)**, and **mixed AI (~40%)** (e.g., neuro-fuzzy, sliding-mode+fuzzy, RL); multimodal sensor fusion (**EMG/EEG/IMU/RGB-D**); intention detection, adaptive assistance, feedback/control | **Upper-limb motor & neuromotor rehab**; majority **wearable (53%)**; operation modes distribution: **active 56%**, **passive 17%**, **assistive 15%**, **resisted 12%**; joints targeted: **elbow 49%**, **wrist 26%**, **shoulder 25%**; mostly lab/clinic prototypes with push toward **lightweight/home/ADL** use | **30 / N/A** | Field trending to lighter, more ergonomic wearables and **sensor-fusion-driven AI**; **ANNs central** (many studies ANN-only), but **clinical validation sparse**; technical gaps include **weight/size, DoF alignment, portability**, and **reporting/standardization**; neuromotor-focused work **limited (~16%)**; calls for **robust trials** and **benchmarks** to ensure real-world impact. | Mixed (AI + technology‑assisted) |
| **Cunha B., et al. (2023)** | **Narrative review/overview** of **home-based shoulder rehabilitation** technologies | **Wearables & sensors**, **rehab robots**, **exoskeletons**, **machine learning/deep learning**, **VR/AR**, **serious games** | **Home programs for shoulder disorders** (musculoskeletal/neuromuscular): remote monitoring, guided exercises, feedback, and telerehab; examples span **assessment→training→progress tracking** | **Narrative—no pooled N** | Maps the state of the art (devices, algorithms, and use-cases); reports generally **positive feasibility/safety** with home systems and **motivation gains** via VR/AR & games; catalogs exemplar **robots/exoskeletons** and **ML pipelines** for exercise detection/scoring; provides **design & ethics guidance** (user-centric UI, real-time feedback, safety limits, privacy/security, clinician oversight) and notes **evidence gaps** (heterogeneous methods, limited RCTs/standardized outcomes). | Mixed (AI + technology‑assisted) |
| **Senadheera et al.** | Scoping review using AI-enhanced methods (topic clustering incl. BERTopic; PRISMA-ScR aligned). | Supervised ML/ANN, DL; NLP; computer vision; BCI; robotics; VR; sensor-based systems (e.g., IMUs). | Adult post-stroke rehabilitation across the care continuum (acute→chronic); settings spanning inpatient/rehab facilities and home. | **704** studies; participants not aggregated (scoping). | Four themes mapped: Impairment; Assisted intervention; Prediction; Imaging/Neuroscience. Upper-limb focus prominent (**310/704; 44%**). Only **16 RCTs (2%)** identified—evidence base dominated by non-RCT designs. Time-linked evolution shows growth in supervised ML, ANN, NLP/DL, and sensor-driven assessments; applications increasingly target tailored therapy and real-world carryover. | Mixed (AI + technology‑assisted) |
| **Mennella C., et al. (2023)** | **Systematic literature review (PRISMA)**; PubMed/Scopus/IEEE Xplore; 2010–May 2022; focus on **decentralized/remote** rehab (monitoring & assistance). | **Supervised & unsupervised ML/DL** (e.g., SVM, RF, KNN, CNN/LSTM/MLP/GCN); **computer vision/pose estimation**; **wearables/IMUs**; Kinect/Leap Motion; exergames & digital coaches/agents. | **Home/tele-rehabilitation** across multiple conditions; tasks: **activity recognition**, **movement classification**, **clinical-status prediction**; supports dose/adherence tracking and real-time feedback. | **35 / N/A** (from **519** records screened; participants not pooled). | Unobtrusive motion capture (wearables/RGB) is **strategic**; most studies are **lab-based** with **healthy subjects**, limiting **generalizability**; overall TRL around **3–4**; calls for **clinical validation at home**, standardized metrics, and reporting. | AI‑enabled (ML-based) |
| **Jawed A.M., et al. (2025)** | **Systematic-style narrative review** (PubMed/Scopus/IEEE/ScienceDirect) on AI/ML across spine diagnosis, surgery, and rehabilitation. | **Deep learning (CNNs)** for imaging; **ML (RF/SVM/predictive analytics)**; **robotic & AR-guided navigation**; **AI wearables/IoT** for posture & recovery; **virtual/tele-physiotherapy** and remote monitoring. | **Spine disorders** (LBP, scoliosis, stenosis, trauma, tumors) across **hospital→home** continuum; **post-op rehab** and prevention emphasized (real-time feedback, adherence tracking). | **Not enumerated / N/A** (mapping review; no pooled participants). | Signals: DL achieves **radiologist-level** detection for vertebral fractures/disc herniations; **robotic navigation** reports **>95%** screw-placement accuracy; AI-optimized imaging may **reduce radiation**; **virtual physio & wearables** support adherence and earlier issue detection. Gaps: **limited datasets**, **bias/ethics**, **real-world validation**, and **integration** with clinical workflows. | Mixed (AI + technology‑assisted) |
| **Ravali R.S., et al. (2022)** | **Systematic review** (Cochrane-guided; PRISMA-reported); searches **Mar 2011–Mar 2021** across PubMed/IEEE/Google Scholar, trial registries, and grey literature; **187** titles/abstracts screened; **48** full texts assessed. | **ML/DL**, computer vision/pose estimation; **wearables/IMUs/EMG** for exercise monitoring; exoskeleton/VR examples; clinical decision-support concepts. | **Pediatric physiotherapy practice** (education + clinical delivery); settings spanning **clinic and home/remote**; musculoskeletal and neuro-oriented tasks. | **Included N not explicitly enumerated**; participants **not pooled**. | Descriptive synthesis (no pooled effects): signals feasibility of **wearable-ML exercise tracking** and **movement/pose analysis**; highlights **pain phenotyping** research, but heterogeneous; emphasizes PT **education reforms** (data, computational, human literacies), and **data standardization/privacy** needs before scale-up. | Mixed (AI + technology‑assisted) |

AD = Alzheimer’s disease
ADL = activities of daily living
AE = adverse event
ALS = amyotrophic lateral sclerosis
ANN = artificial neural network
AUC = area under the (ROC) curve
BCG = ballistocardiography
BCI = brain–computer interface
BI = Barthel Index
BLDA = Bayesian linear discriminant analysis (classifier)
CNS = central nervous system
CNN = convolutional neural network
CP = cerebral palsy
CT/CTA/CTP = computed tomography / CT angiography / CT perfusion
DL = deep learning
DoF = degrees of freedom
DT = decision tree
ECG/PPG = electrocardiography / photoplethysmography
EEG/EMG/sEMG = electroencephalography / electromyography / surface EMG
EHR = electronic health record
ELF-EMF = extremely low-frequency electromagnetic field
e-ASPECTS = automated (AI) Alberta Stroke Program Early CT Score for non-contrast CT brain
FAC = Functional Ambulation Category
FBCCA = filter-bank canonical correlation analysis (SSVEP-BCI feature method)
FES = functional electrical stimulation
FMA-UE = Fugl–Meyer Assessment—Upper Extremity
GCN / ST-GCN = (spatial-temporal) graph convolutional network
HMI = human–machine interface
HRQoL = health-related quality of life
ICT/IoT = information & communications tech. / Internet of Things
IMU / UWB = inertial measurement unit / ultra-wideband
IR* = intelligent robotics (label used by Zhu et al. network meta-analysis)*
IRDS = IntelliRehabDS (physical-rehab movements) dataset
ITR = information transfer rate (BCI)
JBI = Joanna Briggs Institute (scoping-review methodology)
KIMORE = KInematic Assessment of MOvement and Clinical Scores for Remote Monitoring of Physical Rehabilitation (dataset)
LDA = linear discriminant analysis
LLM = large language model
LOS = length of stay
LR = logistic regression
LSTM = long short-term memory (recurrent neural network)
LVO = large-vessel occlusion (ischemic stroke)
MAD = mean absolute deviation/difference
MAE / RMSE = mean absolute error / root mean square error
MBI = Modified Barthel Index
MCA = middle cerebral artery
MCI = mild cognitive impairment
MG = myasthenia gravis
MI (scale) = Motricity Index (strength)
MI (BCI context) = motor imagery
MRI/OCT/PET = magnetic resonance imaging / optical coherence tomography / positron emission tomography
mRS = modified Rankin Scale
NN = neural network
NLP = natural language processing
NPV / PPV = negative / positive predictive value
OSF = Open Science Framework
PD = Parkinson’s disease
PNS = peripheral nervous system
PRISMA / PRISMA-ScR = Preferred Reporting Items for Systematic Reviews and Meta-Analyses / extension for Scoping Reviews
PROSPERO = International Prospective Register of Systematic Review Protocols
RAPID (software) = automated CT/MR perfusion package for ischemic stroke
RF / k-NN = random forest / k-nearest neighbours
RoB 2 = Cochrane Risk-of-Bias tool for randomised trials
RR* = rehabilitation robotics (label used by Zhu et al.)*
RT* = robotic therapy (label used by Zhu et al.)*
SCI = spinal cord injury
SSVEP / P300 = steady-state visual evoked potential / P300 event-related potential
SUCRA = surface under the cumulative ranking curve (network meta-analysis ranking metric)
SVM = support-vector machine
UI-PRMD = University of Idaho—Physical Rehabilitation Movements Dataset
Viz LVO (ContaCT) = Viz.ai tool for LVO detection/notification on CTA
VR/AR = virtual / augmented reality
XAI = explainable artificial intelligence

*Notes on RT/IR/RR: these category labels follow Zhu et al.’s network meta-analysis taxonomy and may differ from other papers’ usage.
